# Functions of* Candida albicans* cell wall glycosidases *Dfg5p* and *Dcw1p* in biofilm formation and HOG MAPK pathway

**DOI:** 10.7717/peerj.5685

**Published:** 2018-09-28

**Authors:** Ryan Mancuso, Jennifer Chinnici, Charlene Tsou, Sujay Busarajan, Raveena Munnangi, Abhiram Maddi

**Affiliations:** 1 Periodontics & Endodontics, State University of New York at Buffalo, Buffalo, NY, United States of America; 2 Periodontics & Endodontics, Oral Biology, State University of New York at Buffalo, Buffalo, NY, United States of America

**Keywords:** *Candida albicans*, *DFG5*, *DCW1*, Hog1, Biofilms

## Abstract

**Background:**

*Candida albicans* is a commensal fungus that inhabits the oral mucosal surface and causes oral and systemic candidiasis. Oral candidiasis most commonly occurs in patients with AIDS, denture wearers and newborn children. Systemic candidiasis occurs mainly in immunocompromised patients and patients admitted to hospitals for prolonged periods. *C. albicans* homologous genes, *DFG5* and *DCW1*, encode for two closely related cell wall proteins with putative glycosyltransferase enzyme activity and C-terminal GPI-anchors. Past studies have shown that individual *DFG5* and *DCW1* mutations are viable but simultaneous deletion of *DFG5* and *DCW1* in *C. albicans* results in lethality. However, the exact functions of these cell wall based enzymes, which represent potential drug targets, are not understood.

**Methods:**

*C. albicans *DFG5*/*DCW1** heterologous and conditional double mutant strains were assessed for growth and biofilm formation in comparison to wild type and parental strains. Cell wall and heat stress susceptibility of the mutant and control strains were assessed using agar spotting assays. Growth was assessed under normal and osmotic stress conditions along with light microscopy imaging. Biofilm dry weight and microscopic imaging analysis of biofilms was performed. Hypha formation in response to serum was analyzed using light microscopy imaging. Western blot analysis of mutant strains and control strains was performed to assess Hog1 basal levels and phosphorylation status.

**Results:**

Analysis of the heterologous mutants indicated that Dfg5p is more important for growth while Dcw1p appeared to play a role in cell wall integrity response. The conditional double mutant was observed to be less resistant to cell wall stress. However, growth of the mutants was similar under control and osmotic stress conditions. The mutants were also able to grow similar to wild type under heat stress. Biofilm formation was reduced in the mutants where *DFG5* was deleted or suppressed. Hyphal morphogenesis was reduced although germ tube formation was observed in the biofilms of the mutant strains. Basal Hog1 protein levels were reduced or absent in the *DFG5* and *DCW1* mutants. However, osmotic stress was able to induce Hog1 protein levels comparable to wild type. Hog1 phosphorylation appeared to be slightly reduced although not significantly. In addition to biofilm assays, serum dose response imaging analysis indicated that hyphae formation in *DFG5* and *DCW1* mutants was defective.

**Conclusions:**

These data indicate that *DFG5* and *DCW1* are required for hyphal morphogenesis and biofilm formation in *C. albicans*. These functions may be regulated via basal Hog1 MAPK which is required for transcriptional regulation of chitin synthesis.

## Introduction

A great majority of all fungal infections in humans are caused by *Candida albicans*, a fungus that occurs in various morphologies including yeast, hyphae, pseudohyphae and chlamydiospores (Veses & Gow, 2009; Sudbery, 2011; Noble, Gianetti & Witchley, 2017). Normally *C. albicans* exists as a commensal in the human body and causes the disease candidiasis under certain conditions. The conditions that result in candidiasis include prolonged antibiotic treatment, immunosuppressive conditions that arise due to genetic disorders or drug therapy, HIV infection, medical and dental prostheses and dry mouth (Bondaryk, Kurzatkowski & Staniszewska, 2013; Cassone & Cauda, 2012). Oral mucosal candidiasis involving the mouth occurs in millions of people worldwide. Disseminated candidiasis occurs in patients who are immunocompromised due to prolonged hospital based treatments and has an associated high mortality rate (Nguyen et al., 2012; Bondaryk, Kurzatkowski & Staniszewska, 2013). Additionally, there is an alarming rise in the incidence of antifungal drug resistance which warrants the need for novel antifungal drugs and drug targets (Bondaryk, Kurzatkowski & Staniszewska, 2013).

Dfg5p and Dcw1p represent homologous cell wall proteins with an N-terminal signal peptide, a GPI-anchor and enzymatic domains for putative glycosidase/mannosidase functions in *C. albicans*. These proteins are primarily located in the cell membrane (Spreghini et al., 2003). The genes that encode these proteins are highly conserved among several ascomycetes fungi and are known to have redundant functions. Past studies in the yeast *Saccharomyces cerevisiae* have shown that simultaneous *DFG5* and *DCW1* deletion is lethal indicating that the cell wall proteins/enzymes encoded by these genes have a critical and redundant role in cell functions (Kitagaki et al., 2002; Kitagaki, Ito & Shimoi, 2004). Similarly studies in *C. albicans* have shown that simultaneous deletion of the *DFG5* and *DCW1* genes is lethal indicating that these cell wall enzymes are critical for normal growth and survival (Spreghini et al., 2003). Additionally, *DFG5* deletion leads to a defect in hyphal formation in alkaline pH. Based on the observation that the expression of *HWP1*, a well-known cell wall gene expressed only in hyphae, was dependent upon the presence of Dfg5p, it has been thought to be involved in signal transduction mechanisms tied into the cell wall integrity pathway (Spreghini et al., 2003). Our studies in *N. crassa* demonstrated that the *dfg5*Δ*/dcw1*Δ double mutant is unable to cross-link cell wall protein into the cell wall matrix, and that the integrity of the cell wall is compromised (Maddi, Fu & Free, 2012). These initial studies in *N. crassa* are significant as this fungus primarily has a hyphae/filamentous morphology and the resulting defect in filamentous growth in the *dfg5*Δ*/dcw1*Δ** mutant indicates the important evolutionary role played by these glycosidases in filamentation (Maddi, Fu & Free, 2012). We have also shown that *C. albicans* Dfg5p and Dcw1p are involved in cell wall protein cross-linking within the cell wall (Ao et al., 2015). In a recent study in *S. cerevisiae* it was observed that Hog1 and Slt2 signaling pathways are affected in the *DFG5* deletion background (Nasution et al., 2015). However, the role of *DFG5* and *DCW1* in biofilm formation and Hog1 signaling mechanisms has not been studied in the pathogenic fungus *C. albicans*.

In *S. cerevisiae*, HOG MAPK signaling pathway mainly functions in response to osmotic stress. However, HOG response also functions in cell wall integrity response and oxidative stress response in *S. cerevisiae* and *C. albicans* (Kruppa & Calderone, 2006). In *C. albicans*, HOG response to stress is not only important for cell survival under stress but also critical for cell survival *in vivo* (Arana et al., 2007). More importantly HOG pathway is required for virulence in a mouse model of candidiasis (Alonso-Monge et al., 1999). In *S. cerevisiae*, the HOG pathway has sensor proteins and histidine kinases, Sln1 and Sho1 that are located in the cell membrane and act as upstream regulators of osmotic stress (Brewster & Gustin, 2014). However, more recent studies have shown that in the pathogenic *C. albicans*, only the Sln1 dependent pathway activates Hog1 MAPK (Román, Nombela & Pla, 2005; Kruppa & Calderone, 2006; Cheetham et al., 2007). However, null mutant of Sln1 in *C. albicans* is not susceptible to osmotic stress (Nagahashi et al., 1998). Investigation of two other histidine kinases Nik1 and Hk1, along with Sln1 found that all three histidine kinanses functioned independent of Hog1 (Román, Nombela & Pla, 2005). Interestingly, Sln1, Nik1 and Hk1 deletion mutations in *C. albicans* lead to defects in hyphal morphogenesis (Yamada-Okabe et al., 1999). This clearly shows that in *C. albicans*, the MAPK signaling circuits are much different and more complicated than *S. cerevisiae* and this may provide an evolutionary advantage to this opportunistic pathogen. However, the exact physical mechanism of osmosensing or phenotypic switching is not clearly understood (Brewster & Gustin, 2014). We hypothesized that the cell wall mannosidases, Dfg5p and Dcw1p, act as the mechanical switch between HOG osmotic response and hyphal morphogenesis depending on environmental cues.

The study of *DFG5* and *DCW1* in *C. albicans* by Spreghini et al. (2003), utilized several single mutant strains and double mutant strains including homozyogous, heterozygous and conditional mutants. In that study, the single mutants of *DFG5* and *DCW1* were well characterized and were found to be viable. Only the *dfg5*Δ*/dfg5*Δ** single mutant was found to have a filamentous growth defect at alkaline pH, indicating that these genes have redundant and compensatory functions. However, the exact functions of Dfg5p and Dcw1p are still not known. In our study we utilized the heterozygous and the conditional double mutants of *DFG5* and *DCW1* in order to characterize the redundant functions of these genes. These heterozygous and conditional mutants include the ES1, ES195 and the ES195 conditional mutant (Table 1). The ES195 conditional mutant especially is powerful in that it takes the mutant strain to the very brink of deletion without causing lethality. To our knowledge this is the first study to determine the role of *C. albicans DFG5* and *DCW1* in biofilm formation as well as in Hog1 MAPK signaling mechanisms.

**Table 1:** *Candida albicans* strains used in this study.

**Group**	**Strain**	**Genotype**
**Control 1**	SC5314	Wild type
**Control 2**	BWP17	Wild type parental
**Control 3**	DAY185	Wild type parental (URA Reintegrated)
**Test 1**	ES1	*DFG5* homozygous deletion strain with one copy of *DCW1*
**Test 2**	ES195	*DFG5* and *DCW1* deletion with 1 copy of *DFG5* expressed using a Methionine promoter
**Test 3**	ES195+Met/Cys	Conditional *DFG5/DCW1* mutant 5mM Methionine and 2mM Cysteine added for 1 h to media for 85% *DFG5* repression

## Materials & Methods

### Strains and growth conditions

For wild type, SC5314 strain of *Candida albicans* was used. In addition, parental WT strains BWP17 (URA- or uridine auxotroph) and reintegrated strain DAY185 (URA+) were used in some experiments. These strains along with the test strains, ES1 and ES195, were provided as a kind gift from Dr. Aaron Mitchell (Carnegie Mellon University, Pittsburgh, PA, USA) and have also been deposited at the fungal genetics stock center (FGSC). Other strains—*hog1*Δ**, *sln1*Δ**, *nik1*Δ** and *hk1*Δ** were obtained from the *Candida albicans* kinase plates (Mitchell Kinase plates 1 & 2, FGSC). The ES1 and ES195 strains have been previously described (Spreghini et al., 2003). ES1 has a *dfg5*Δ*/dfg5*Δ**::*dcw1*Δ*/DCW1* genotype. ES195 has a *dfg5*Δ*/dfg5*Δ**::*dcw1*Δ*/dcw1*Δ** genotype, but also contains an ectopic copy of the *DFG5* coding region with the upstream *MET3* regulatory elements. ES195 is viable when grown in the absence of methionine and cysteine (when the chimeric copy of *DFG5* is expressed), but stops growing when the chimeric gene is turned off by adding methionine and cysteine to the medium. The strains were cultured in Yeast Nitrogen Base (YNB) medium with ammonium sulfate and 2% glucose adjusted to pH 7. Synthetic complete supplement mixture (MP Biomedicals, Santa Ana, CA, USA) was added as aminoacid supplement to YNB. A total of 5 mM Methionine and 2 mM cysteine were added to the medium for ES195 strain for conditional repression (85%) of the chimeric *MET3::DFG5* gene to generate a Dfg5p-deficient condition.

### Spotting and growth assays with and without 1 M sorbitol

WT (SC5314), WT (BWP13), ES1 and ES195 were plated in YNB with 5 mM methionine and 2 mM cysteine and with or without 1 M Sorbitol. Plates were incubated at 30 °C for 48 h. Spotting assays were performed and images were taken at 24 and 48 h. For assessment of osmotic stress in liquid cultures wild type, parental and mutant strains strains were cultured in YNB or YNB with 1 M sorbitol for 24 h and OD600 readings were obtained at 0, 2, 4, 6, 8, 12 and 24 h. To the cultures containing ES195 conditional mutant, methionine and cysteine were added, for a final concentration of 5 mM and 2 mM respectively, to shut off the ectopic copy of *DFG5*. The growth assays were performed in triplicates on at least three different days. The OD600 readings were graphed against time in Microsoft Excel.

### Cell wall stress tests

To determine if the mutants were affected in the synthesis of the cell wall, growth tests in the presence of cell wall stress reagents were carried out as described previously (Ao et al., 2015). Overnight cultures of WT, ES1, and ES195 strains were inoculated from frozen stocks in YNB and cultured overnight at 30 °C with shaking at 225 RPM. The cell counts for the overnight cultures were determined using a hemocytometer. The cells were transferred to fresh YNB (pH 7) for a final concentration of 1 × 10^6^ cells/mL. To the tubes containing ES195, methionine and cysteine were added, for a final concentration of 5 mM and 2 mM respectively, to shut off the ectopic copy of *DFG5*. Cultures were incubated at 30 °C for 1 h with shaking at 225 RPM. A 1:10 dilution series was made with each culture. 5 ul each of the undiluted, 1:10, 1:100, and 1:1,000 dilution samples were spotted onto YNB pH7 plates containing one of the following cell wall stress agents: Calcofluor White (20 µg/mL), Caspofungin (0.25 µg/mL), Congo Red (1 µg/mL), 100 µg/mL SDS (100 µg/mL), or Sorbitol (1 M). Concentrations of stress agents were based on MIC values as described previously (Ao et al., 2015; Nikolaou et al., 2009; Heilmann et al., 2013). In addition, just for the 1 M sorbitol experiments WT (SC5314), WT (BWP13), ES1 and ES195 were plated in YNB with 5 mM methionine and 2 mM cysteine. Plates were incubated at 30 °C for 48 h. Spotting assays were also done under heat stress at 39 °C. Images were taken at 24 and 48 h. The ability of the ES1 and ES195 strains to grow in the presence of these cell wall stress agents was observed and compared with the growth of the WT strain.

### Biofilm biomass analysis

Overnight cultures of WT, ES1, and ES195 strains were inoculated from frozen stocks in YNB and cultured overnight at 30 °C with shaking at 225 RPM. The cell counts for the overnight cultures were determined using a hemocytometer. The cells were transferred to fresh YNB (pH 7) supplemented with 20% FBS for a final concentration of 1 × 10^6^ cells/mL. To the tubes containing ES195, methionine and cysteine were added for a final concentration of 5 mM and 2 mM respectively, to shut off the ectopic copy of *DFG5*. The cultures were transferred to six well polystyrene culture plates (Falcon, Corning, NY), 2 mL per well, and incubated at 37 °C statically for 24 hrs. After incubation, the media over the resulting biofilms was carefully removed and the biofilms were washed once with 1X PBS. They were then removed with a pipette and additional 1X PBS to pre-weighed microfuge tubes as described before (Li et al., 2013). The samples were centrifuged to pellet the cells and remove most of the liquid to facilitate drying. The sample tubes were opened and placed in a desiccator jar with anhydrous calcium chloride used as the desiccant. Dry cell mass was quantified after ∼3 days using and analytical weighing scale (Mettler Toledo).

### Microscopy imaging of biofilms

Cultures of wild type and mutant *C. albicans* strains were inoculated from frozen stocks and grown overnight. The concentrations of the overnight cultures were determined and then used to inoculate a culture with the starting concentration of 1 × 10^6^ cells/ml for each strain in a total volume of 6 ml of YNB supplemented with 20% Fetal Bovine Serum (FBS). Methionine and Cysteine, at a final concentration of 5 mM and 2 mM respectively, were added to ES195 to shut off the ectopic copy of *DFG5*. Each culture was transferred to uncoated 6-well polystyrene culture plates (2 ml/well) and incubated statically for 24 h at 37 °C. After incubation, the media was carefully removed and the biofilms were washed once with 1x PBS. Images of the biofilms were taken using a Nikon Eclipse TE2000-U microscope and the Spot Advance software Version 4.0.4 at a total magnification of 200×. The 20× objective was used for the imaging, the oculars were 10×.

### Microscopy imaging under osmotic stress with 0.4 M NaCl

Cultures of wild type and mutant *C. albicans* strains were inoculated from frozen stocks and grown overnight. The concentrations of the overnight cultures were diluted 1:10 in fresh YNB with or without 0.4 M NaCl for a concentration of 1 × 10^7^ cells/ml for each strain in a total volume of 1 ml. Methionine and Cysteine, at a final concentration of 5 mM and 2 mM respectively, were added to ES195 to shut off the ectopic copy of *DFG5*. Cells were incubated statically at 37 °C for 1 h. Drops of each culture were put on slides and images were taken using a Nikon Eclipse TE2000-U microscope and the Spot Advance software Version 4.0.4 at a total magnification of 200×. The 20× objective was used for the imaging, the oculars were 10×.

### Analysis of Hog1 MAPK basal levels and phosphorylation status

Overnight cultures of WT (SC5314), WT parental (BWP17), ES1, and ES195 strains were inoculated from frozen stocks in YNB with complete supplement mixture (CSM) and cultured overnight at 30 °C with shaking at 225 RPM. The cell counts for the overnight cultures were determined using a hemocytometer. The overnight cultures were added to fresh YNB for a total volume of 100 mL for each strain. The cells were allowed to grow to about mid-log phase (around 5 × 10^7^ cells/mL). To induce phosphorylation of Hog1 and assess levels of activated protein, NaCl was added to each culture for a final concentration of 0.4 M and allowed to incubate with shaking for 5 min. Control cultures (no NaCl added) were used to assess total Hog1 levels. Methionine (5 mM) and Cysteine (2 mM) were added to ES195 to shut off the ectopic copy of *DFG5*. The cells were harvested by centrifugation at 5,860× g for 10 min and washed twice with cold 1X PBS. Cell pellets, approximately 300 ul, were transferred to microfuge tubes in order to pulverize them and prepare cell extracts. Two volumes of additional 1X PBS was added to each sample (600 ul) along with 1 volume of 0.5 mm zirconium oxide beads (300 ul) (Next Advance, Troy, NY, USA). Cells were then pulverized in a Bullet Blender Storm 24 (Next Advance, Troy, NY, USA) following the recommended settings for *C. albicans* (Speed 10 for 3 min). The samples were placed on ice for 5 min and subjected to one more cycle in the Bullet Blender. After pulverizing, the samples were centrifuged for 2 min at 12,000× g. The supernatants, containing the cell extracts, were transferred to new tubes and subjected to a DC Protein Assay (BioRad, Hercules, CA, USA) to determine protein concentration. The cell extract protein (10 µg) of non-induced cells were subjected to SDS PAGE gel electrophoresis for protein separation. The protein gels were the subjected to Western transfer to PVDF and then Western blot analysis using anti-pHog1 antibody (Cell Signaling Technology, Danvers, MA, USA) for measuring phosphorylated Hog1 was performed as described previously (Adhikari & Cullen, 2014; Cullen, 2015). Anti-Rabbit HRP conjugated secondary antibody was used to detect the primary antibody. The blot was then stripped and then reprobed using the anti-Hog1 antibody (Santacruz Biotechnology, Santacruz, CA) for detecting total Hog1. An ECL Clarity Kit (BioRad, Hercules, CA, USA) was used with Image Lab 5.2.1 software and the Gel Doc XR+ (BioRad, Hercules, CA, USA) to image the western blots and determine band intensities for later analysis. Band intensities for phosphorylated Hog1 in ES1 and ES195 and WT (SC5314) strains were compared to that of the total Hog1 for triplicate experiments on the same blot using ECL analysis. Anti-pHog1 blot for WT (SC5314) and WT parental (BWP13) was run to determine if phosphorylation of Hog1 was affected in the parental strain. Coommassie staining of the blot was performed for checking loading controls. The blot was then destained prior to Western blot analysis.

### Hypha formation in response to serum

Overnight cultures of WT, ES1, and ES195 strains were inoculated from frozen stocks in YNB and cultured overnight at 30 °C with shaking at 225 RPM. The cell counts for the overnight cultures were determined using a hemocytometer. The cells were transferred to fresh YNB alone and YNB supplemented with 4%, 10% and 20% Fetal Bovine Serum (FBS) (Seradigm) for a final concentration of 2 × 10^7^ cells/mL. To the tubes containing ES195, methionine and cysteine were added for a final concentration of 5 mM and 2 mM respectively, to shut off the ectopic copy of *DFG5*. The tubes were incubated statically at 37 °C for 4 h in six well plates. Light microscopy imaging was performed using a Nikon Eclipse TE2000-U microscope and the Spot Advance software Version 4.0.4 at a total magnification of 200×.

### Statistical analysis

Statistical analysis was performed using Microsoft Excel on a Windows operating system. Each experimental group had a triplicate of samples. Experimental groups were compared using Student’s *t*-test for two samples assuming equal variances. A *p*-Value of <0.01 was considered significant.

## Results

### *DFG5* and *DCW1* heterologous mutations lead to variable growth defects

**Figure 1:**
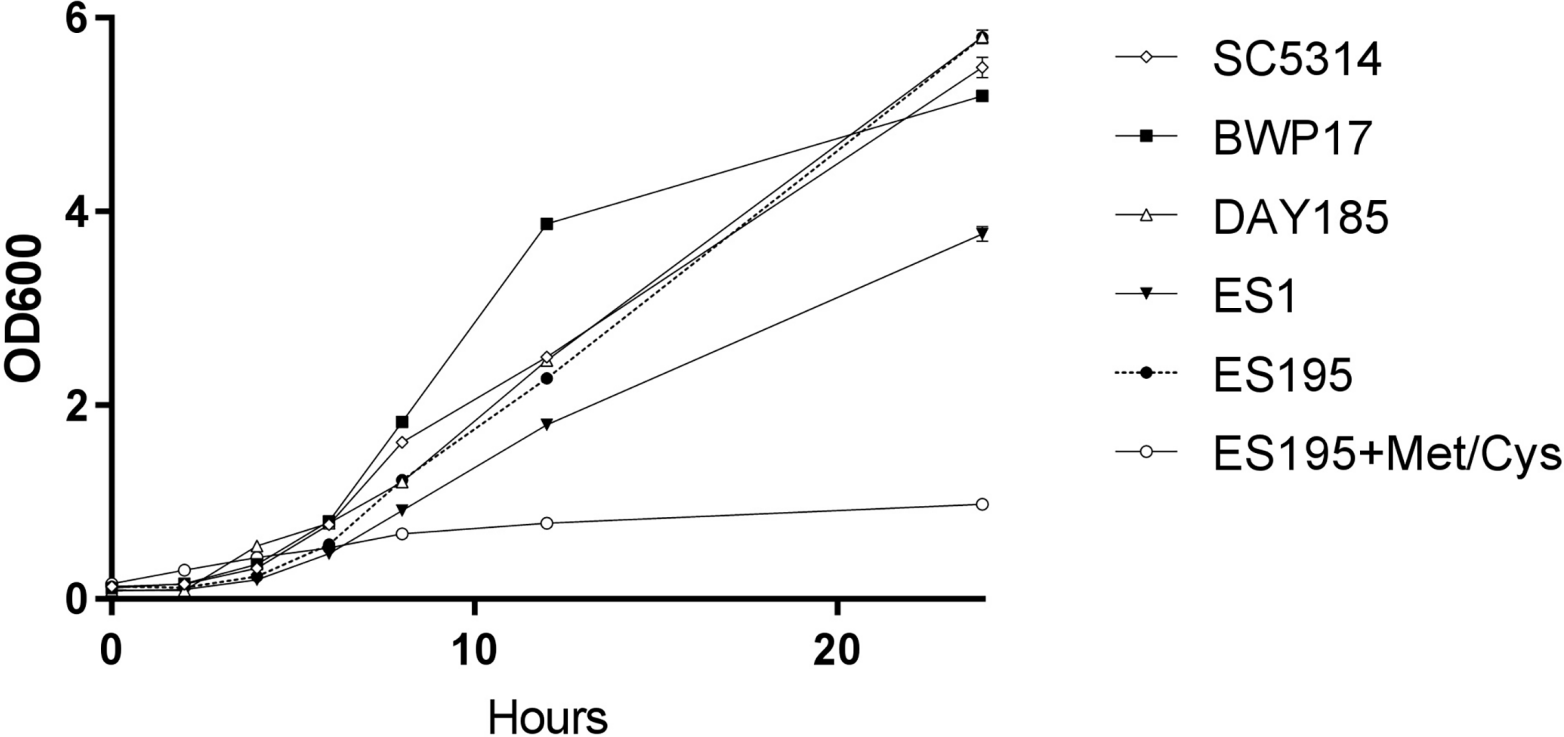
*DFG5* and *DCW1* heterologous mutants have growth defects. Strains were cultured in YNB at 30 degrees Celsius with shaking over 24 h. OD600 readings were obtained at 0, 2, 4, 6, 8, 12 and 24 h time points. To the cultures containing ES195 conditional mutant, methionine and cysteine were added for a final concentration of 5 mM and 2 mM respectively, to shut off the *Met3DFG5* copy.

A 24 h culture of the wild type and mutant strains revealed that the mutant strains ES1(*dfg5*Δ*/dfg5*Δ**::*dcw1*Δ*/DCW1*) had a slight growth defect while ES195 (*dfg5*Δ*/dfg5*Δ**::*dcw1*Δ*/dcw1*Δ**/*MET3::DFG5*) with methionine and cysteine had a major growth defect. On the other hand, ES195 without methionine and cysteine grew normally and comparably to the wild type strains. This indicates that there is a variable rescue of growth when these genes are deleted with one copy of the other gene present. This data indicates that *DFG5* may be required for growth more than *DCW1* despite their redundant functions. On the other hand, the conditional repression (85%) of *DFG5* in ES195 + Met/Cys leads to a severe defect, confirming that the simultaneous deletion of both *DFG5* and *DCW1* is lethal. This data also shows that the genetic background of the parental wild type strain (BWP17) and the reintegrated strain (DAY185) does not affect their growth rate in comparison to the wild type strain (SC5314) (Fig. 1).

### *DFG5* and *DCW1* heterologous mutants have a differential response to cell wall stress and heat stress

**Figure 2:**
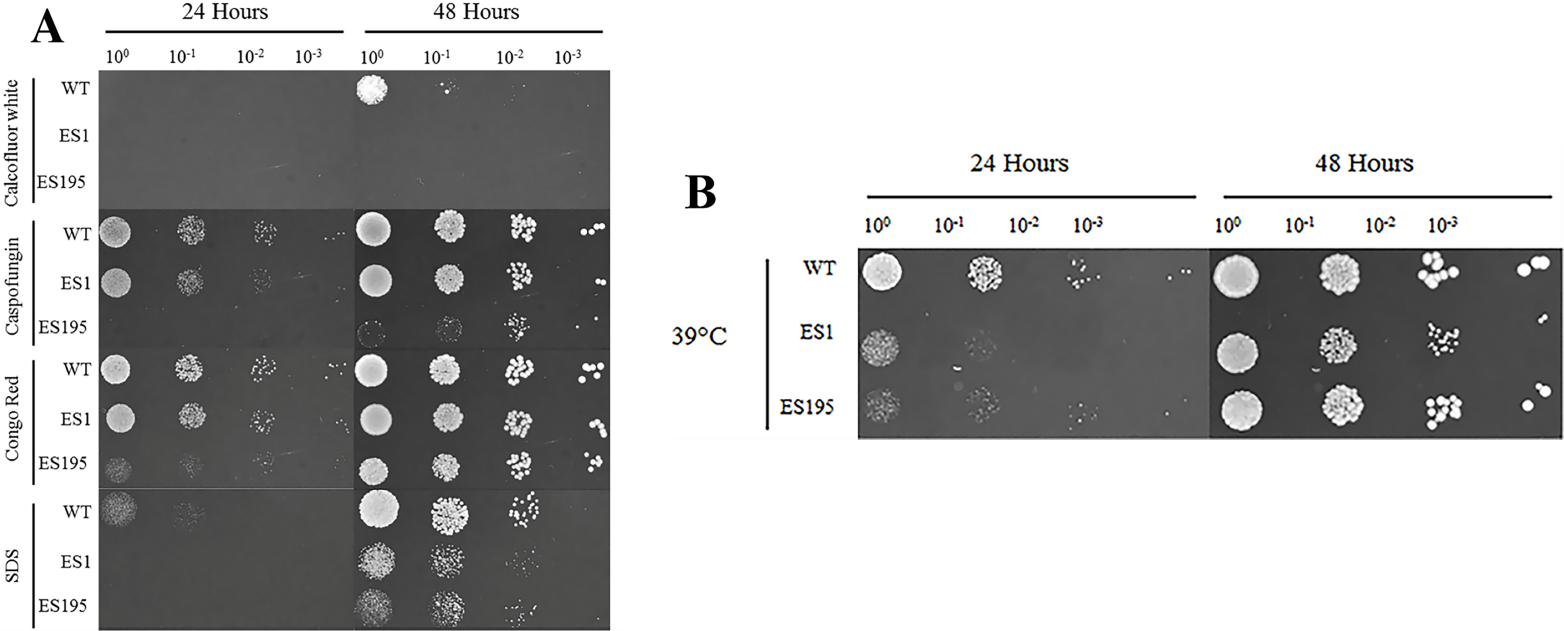
*DFG5* and *DCW1* mutations increase susceptibility to cell wall stress but are not affected by elevated heat stress. WT and mutant strains were spotted on YNB agar in the presence of (A) cell wall stress agents or (B) elevated temperature.

The oral cavity has a pH of 7 generally. In this experiment we performed spotting assays to test the ability of ES1 and ES195 mutant strains in withstanding cell wall stress and temperature stress at pH7. We did not include the conditional mutant ES195+Met/Cys here as it has a severe growth defect and is unable to withstand cell wall stress. The ability of the mutants and the WT (SC5314) to withstand cell wall stress was determined using calcoflour white, caspofungin, congo red and SDS. The ES1 mutant behaved very similar to WT and was able to recover well by 48 h in all agents except for calcofluor white. On the other hand, the ES195 mutant strain was affected by all cell wall stress agents including calcoflour white, caspofungin, congo red and SDS at 24 h (Fig. 2A). The ES195 strain was able to recover by 48 h only in the presence of Congo Red. Generally, fungi respond to cell wall stress by activating the cell wall integrity response. Thus, although there is an increased susceptibility of the mutant strains initially at 24 h, they tend to recover by 48 h. The differences between the two mutant strains indicates that *DFG5* and *DCW1* may have different functions. The mutant strains were also affected by temperature stress (39 °C) at 24 h but they recovered completely by 48 h (Fig. 2B). The better recovery of ES1 and the increased susceptibility of ES195, to cell wall stress indicates that *DCW1* may play a more important role in cell wall integrity.

**Figure 3:**
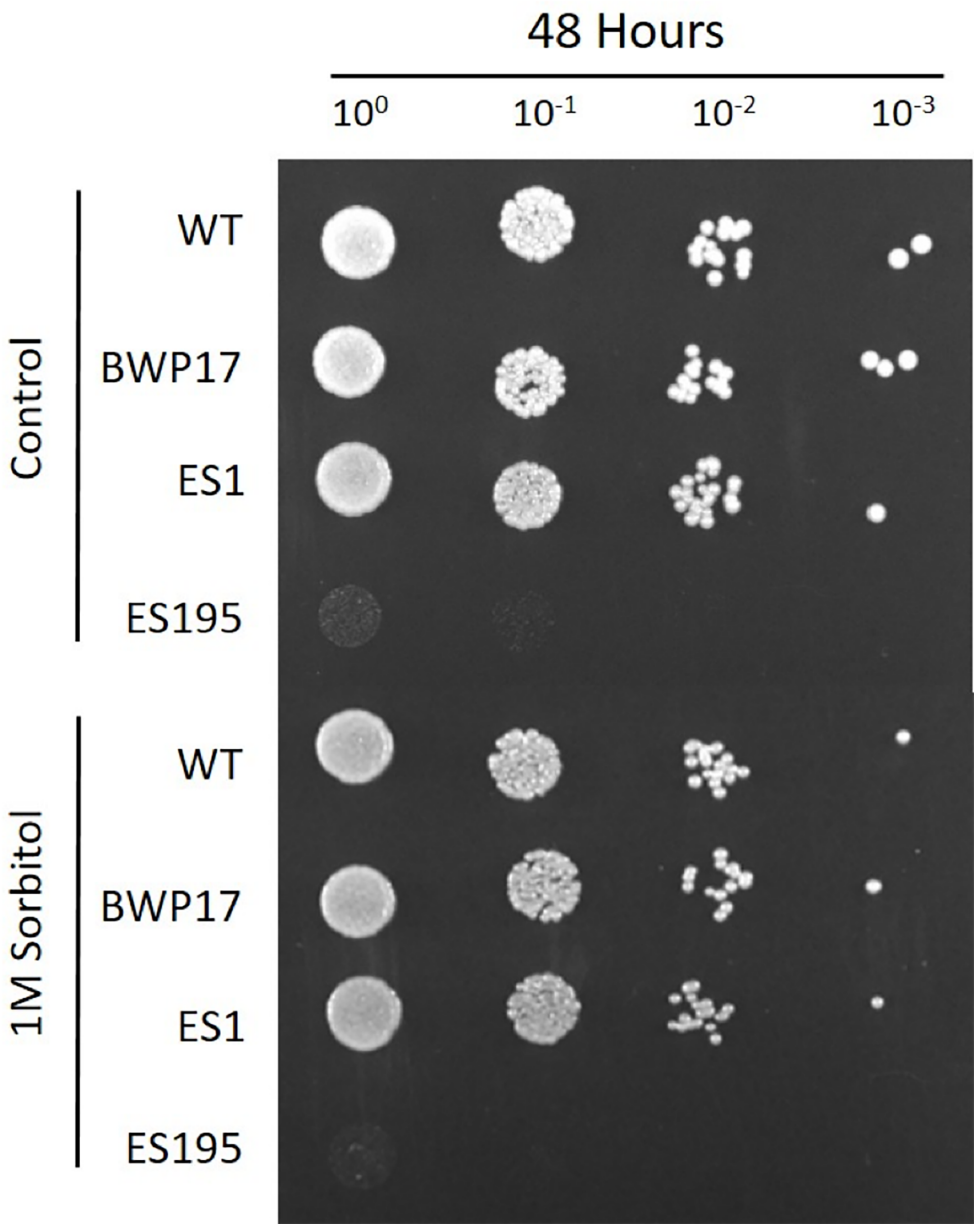
*DFG5* and *DCW1* mutations do not affect osmotic stress response. Strains were cultured for at 30 degree Celsius for 24 h in YNB agar (control) or YNB agar with 1M sorbitol to examine growth under osmotic stress. 2 mM Cysteine and 5 mM Methionine was added to YNB for both control and stress plates to test the ES195 conditional mutant.

**Figure 4:**
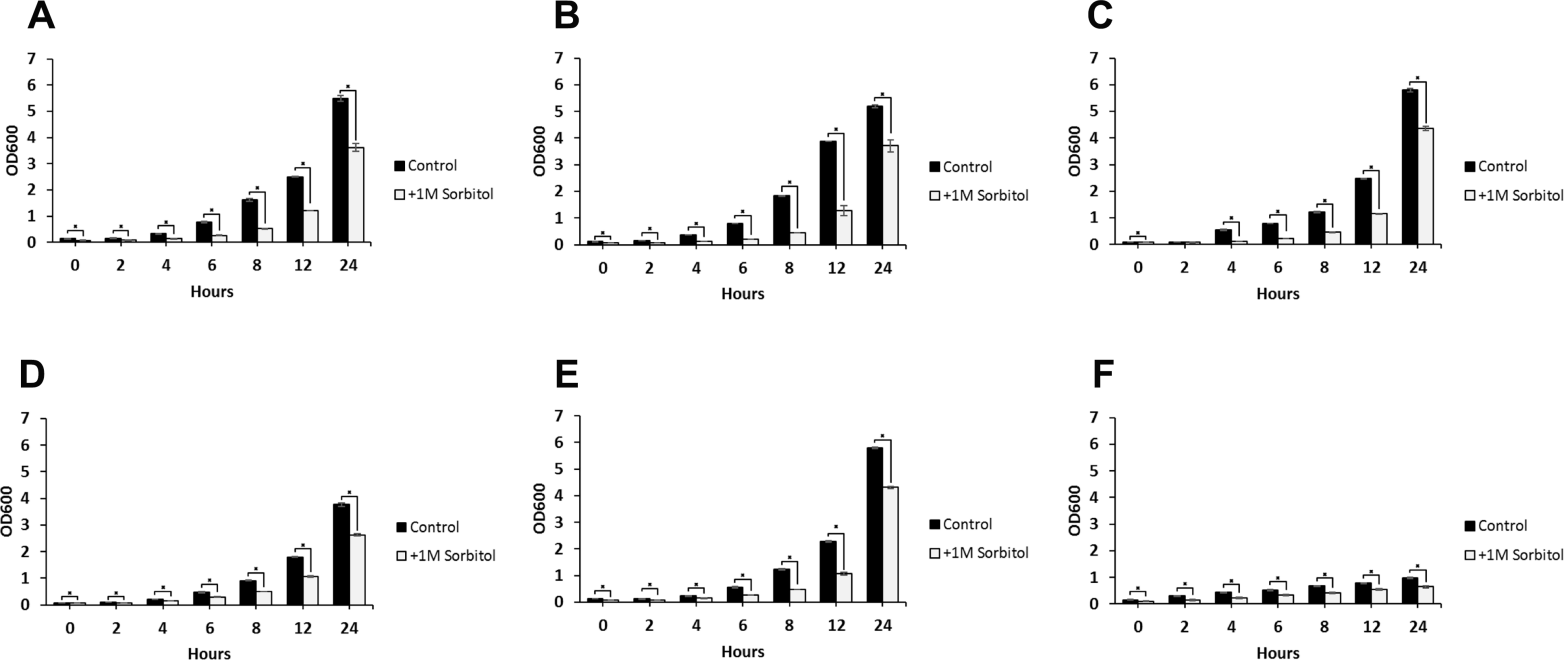
Growth rates of *DFG5* and *DCW1* mutants under osmotic stress. (A) WT (SC5314), (B) BWP17, (C) DAY185, (D) ES1, (E) ES195 and (F) ES195 + Met/Cys. Strains were cultured for at 30 degree Celsius for 24 h in YNB (control) or YNB with 1M sorbitol to examine growth under osmotic stress. Statistical analysis was done using Student’s *t*-test (*p* < 0.01).

### *DFG5* and *DCW1* mutants are unaffected by osmotic stress

The ability to withstand osmotic stress is critical for cell survival. Osmotic stress response is regulated by HOG MAPK pathway in *S. cerevisiae*. The response to osmotic stress can be tested by growing cells in the presence of 1 M sorbitol. Spotting assays were performed on YNB agar plates for wild type and mutant strains, in the presence or absence of 1 M sorbitol (Fig. 3). The YNB plates were prepared with methionine and cysteine. Spotting assays revealed that the growth of the mutants was similar under control (no stress plate) and stress (1 M sorbitol) conditions. Additionally, we performed growth assays to compare the growth rates of WT (SC5314), WT parental (BWP17), URA+ parental strain (DAY185), ES1, ES195 and ES195+Met/Cyst (conditional mutant) in the presence or absence of 1 M sorbitol over a 24 h period. After 24 h, both WT and mutant strains appeared to have significantly decreased growth rates in the presence of 1 M sorbitol as compared to control (no stress) cultures (Fig. 4). The ES195 strain without Met+Cys seemed to grow normally compared to WT strains. Also the ES1 and the ES195  + Met/Cys (conditional mutant) strains seemed to have similar recovery rates in the presence of 1 M sorbitol as compared to WT strain. This data indicates that the simultaneous deletion of *DFG5* and *DCW1* does not affect the ability to overcome osmotic stress, but the recovery to stress could possibly be slower due to a growth defect. Additionally, this experiment further clearly shows that the parental strain BWP17 grows similar to WT (SC5314) strain in 1 M sorbitol and thus is not affected by its parental genetic background.

### *DFG5* and *DCW1* mutants exhibit abnormal phenotypes

**Figure 5:**
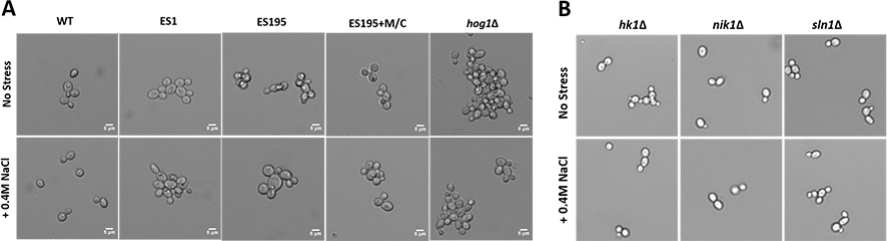
Imaging analysis of *DFG5/DCW1* mutants under osmotic stress. (A) Wild type and *DFG5/DCW1* mutant strains and (B) Histidine kinase mutants, were incubated in YNB (control) or YNB with 0.4M NaCl for 1 h to induce osmotic stress. Imaging analysis was done using light microscopy at 200× magnification.

Light microscopic imaging of mutants revealed abnormal phenotypes under control conditions. *DFG5* and *DCW1* mutants, ES1, ES195 and ES195 conditional mutant appeared to have a cell separation defect (Fig. 5A). This phenotype was somewhat similar to the *hog1*Δ** mutant which appeared to have a much more severe cell separation defect (Fig. 5A). Additionally, under osmotic stress conditions, the ES195 mutant appeared to have an enlarged cell phenotype. On the other hand, the mutants of the three histidine kinases, *hk1*Δ, *nik1*Δ** and *sln1*Δ** did not appear to have any abnormal phenotypes under normal as well as osmotic stress conditions (Fig. 5B).

### *DFG5* and *DCW1* mutations affect basal Hog1 MAPK levels

**Figure 6:**
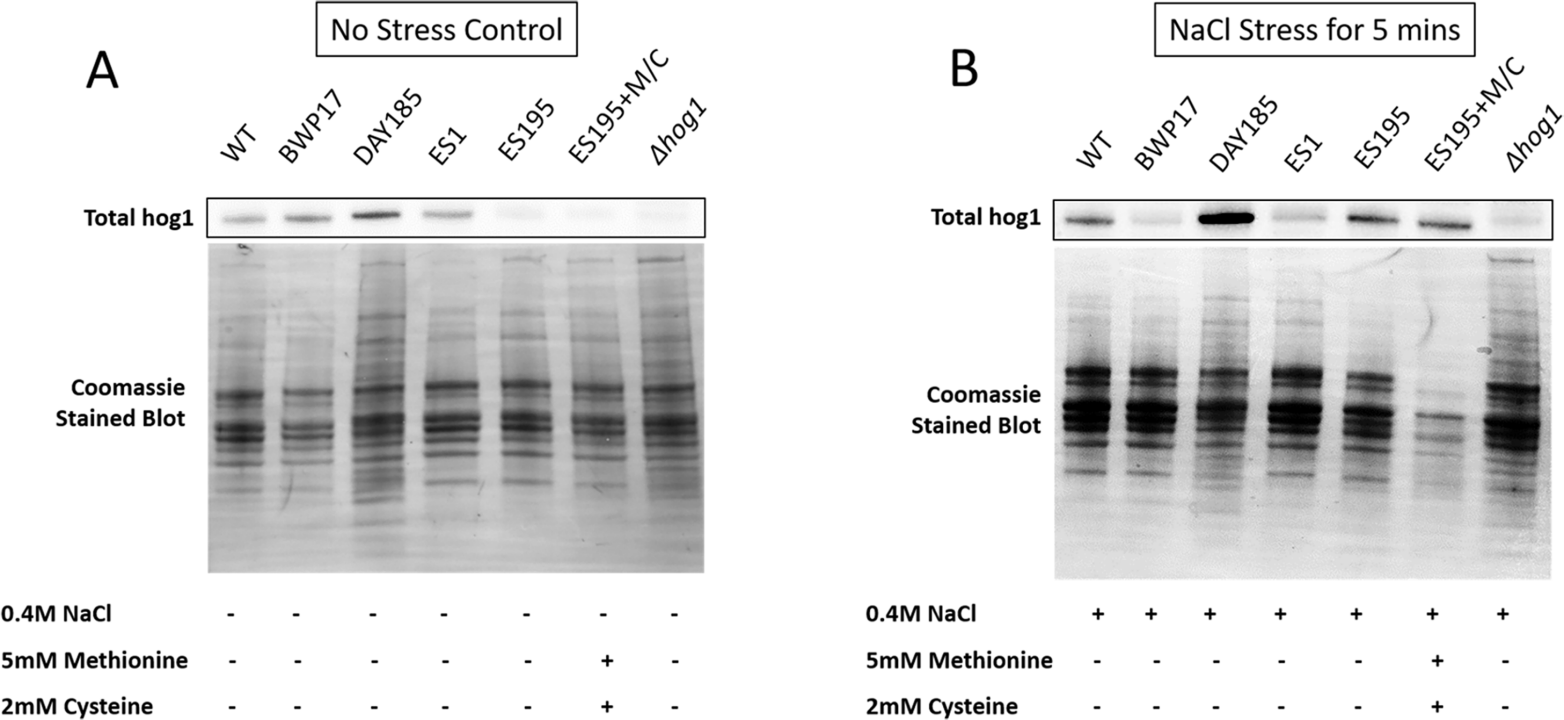
*DFG5* and *DCW1* are required for basal Hog1 levels. Western blot analysis was performed using anti-Hog1 antibody to detect whole Hog1 (non-phosphorylated) under (A) non-stress and (B) osmotic stress conditions.

**Figure 7:**
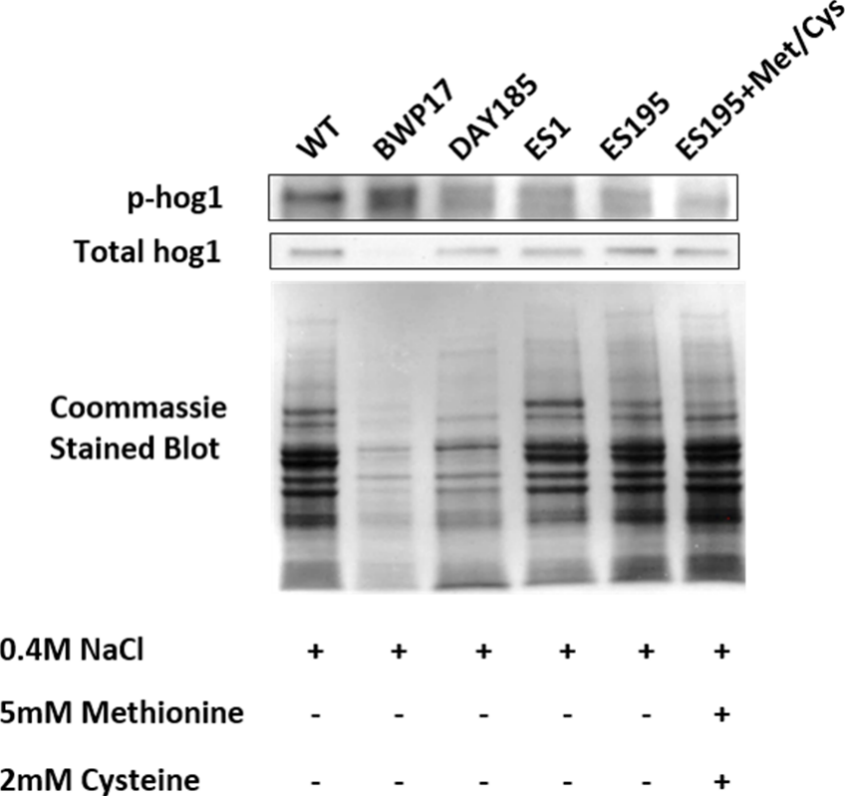
*DFG5* and *DCW1* heterologous mutations affect Hog1 phosphorylation. Phospho Hog1 and whole Hog1 Western blots. Coomassie staining of the blot was performed loading controls, prior to antibody detection.

Western blot and ECL analyses of parental wild type (BWP17), reintegrated strain (DAY185) and WT (SC5314) revealed that basal Hog1 MAPK levels were not affected in these strains under non-stress conditions (Fig. 6A). This indicates that the genetic manipulation of the parental strains did not affect Hog1 MAPK levels in these strains. However, in the mutant strains Hog1 levels appeared to be reduced in the ES1 mutant and absent in the ES195 and ES195 conditional mutants, as compared to wild type (Fig. 6B). Here we utilized the *hog1*Δ** mutant as a negative control for this experiment. However, upon osmotic stress following the addition of 0.4 M NaCl, hog1 MAPK appeared to be induced even in the *DFG5* and *DCW1* mutants. This indicates that Hog1 regulation in response to osmotic stress may occur independently of *DFG5* and *DCW1* in *C. albicans*. Additionally, we also examined the phosphorylated Hog1 MAPK levels in mutants as compared to wild type under stress conditions. The phosphorylated Hog1 MAPK levels for the *DFG5* and *DCW1* mutants did not appear to be significantly reduced (Fig. 7). These results indicate that Hog1 phosphorylation is independent of the *DFG5* and *DCW1* mutations under osmotic stress conditions.

### *DFG5* and *DCW1* mutations affect biofilm formation

**Figure 8:**
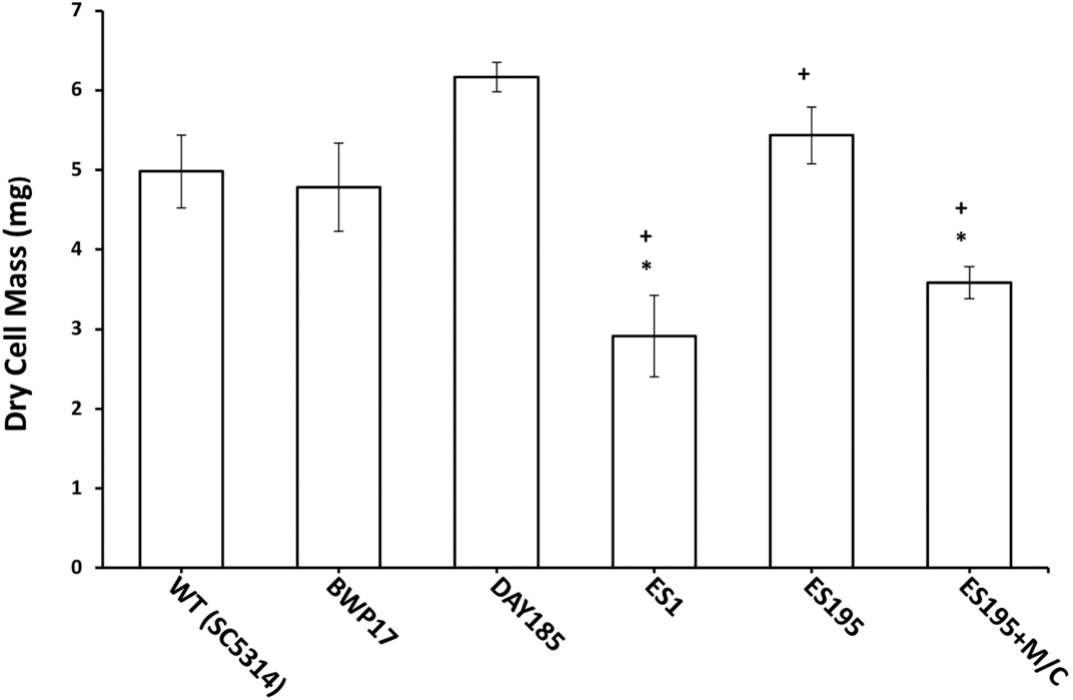
Biofilm formation is affected in Dfg5p and Dcw1p mutants. Biofilm formation was significantly lower for ES1 mutant and ES195+M/C conditional mutant (*p* < 0.01) as compared to WT (SC5314). However, biofilm formation for ES195 mutant was similar to WT (SC5314).

**Figure 9:**
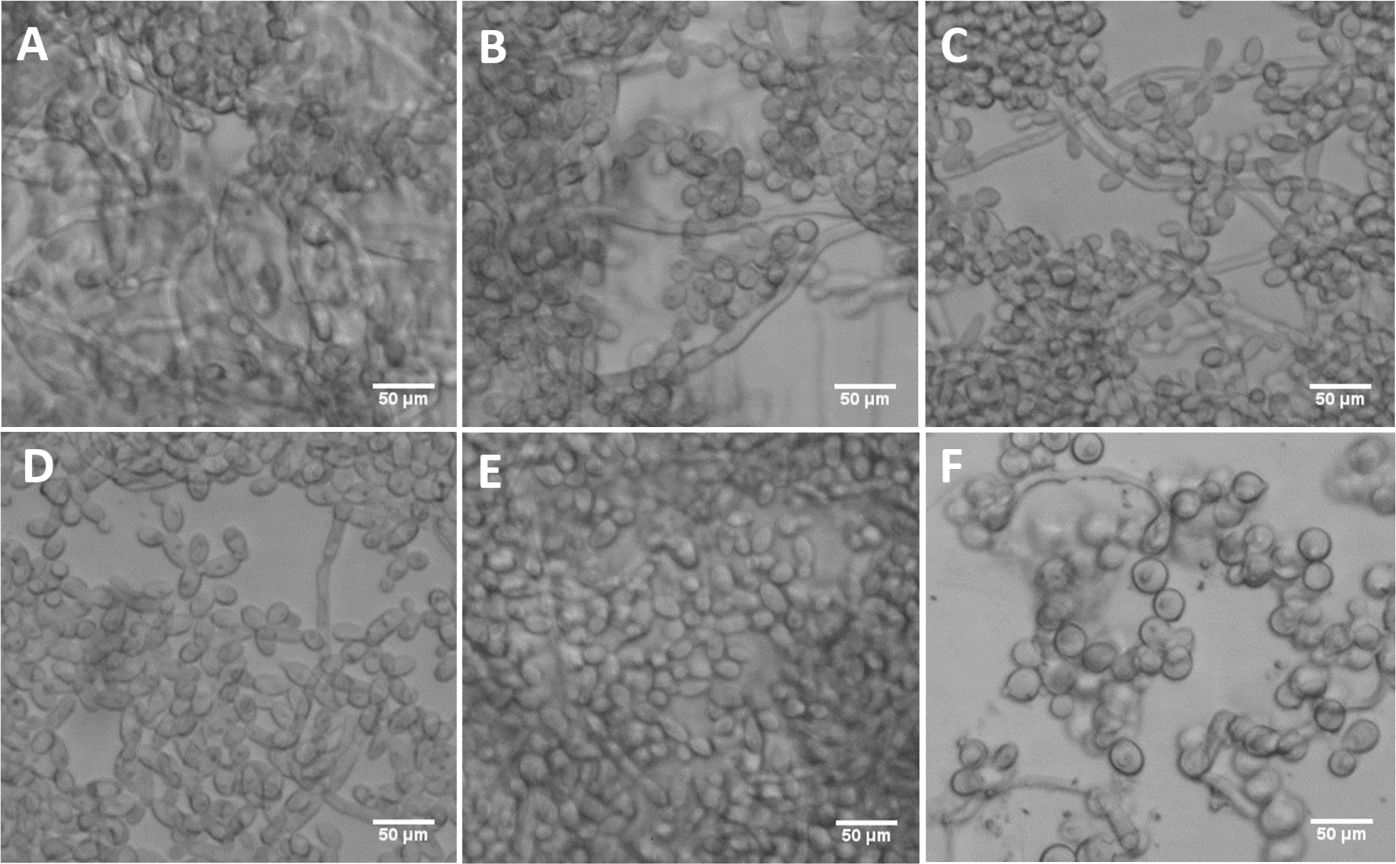
*DFG5* and *DCW1* mutant biofilms have morphological defects. (A) WT (SC5314), (B) BWP17, (C) DAY185, (D) ES1, (E) ES195 and (F) ES195+Met/Cys. Biofilms of wild type and mutant strains were cultured for 24 h. Microscopic imaging analysis of the biofilms was performed using light microscopy at 200× magnification. Hyphal morphogenesis appeared to be dramatically reduced in the ES1, ES195 and ES195+Met/Cys mutants. ES195+Met/Cys conditional mutant has enlarged cells, prominent nuclei, cell separation defect and pseudohyphal formation.

The mutant strains, ES1 and ES195 + Met/Cys (conditional mutant) produced significantly less biofilm as compared to WT and parental strains (Fig. 8). However, the ES195 strain is able to form biofilm biomass similar to the WT strain. In order to further examine the biofilms of these mutants, we performed light microscopy imaging of their 24 h biofilm cultures (Fig. 9). This experiment showed that the mutants have abnormal morphology in biofilms. The ES1, ES195 and ES195 conditional mutants generally had reduced hyphal morphogenesis as compared to controls (Fig. 9). The ES195(*dfg5*Δ*/dfg5*Δ**::*dcw1*Δ*/dcw1*Δ**/*MET3::DFG5*) conditional strain which has 85% suppression of the ectopic copy of *DFG5* was observed to have a very distinct morphology. In this mutant, biofilm was sparse supporting a reduced biomass. The cells were generally enlarged and they were clumpy indicating a cell separation defect. Additionally, pseudohyphae formation was observed. This phenotype indicates that there is a possible cell cycle arrest where the cells are unable to progress to fully developed hyphae. However, the overall biofilm mass was similar between ES1 and the ES195 conditional mutant although the latter has a much more severe growth defect. There is a more severe defect in hyphal morphology for both strains due to a possibly decreased Hwp1 expression resulting from Dfg5p deletion as shown previously (Spreghini et al., 2003). This data suggests that *DFG5* and *DCW1* mutations affect biofilm formation.

### *DFG5* and *DCW1* mutations lead to defects in hyphae formation

**Figure 10:**
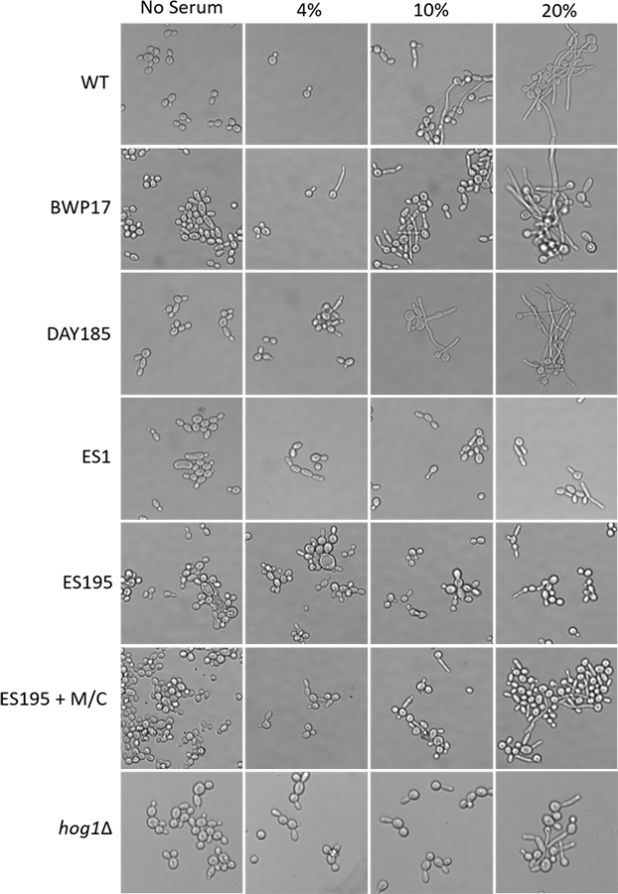
Dfg5 and Dcw1 are required for hyphal morphogenesis. Strains were incubated in YNB with serum at various concentrations (0%, 4%, 10% & 20%). 2 × 10^7^ cells were incubated in six well plates with YNB with serum at 37 degrees Celsius for 4 h statically. The cells were transferred to a glass slide and light microscopic imaging was performed at 200× magnification.

In order to assess hypha formation, the wild type and mutant strains were subjected to increasing serum dosage and light microscopy imaging analysis was performed. Based on this analysis, ES1 mutant appeared to have a severe defect in hyphae formation even at 20% serum. On the other hand, ES195 and ES195 conditional mutant showed pseudohyphae formation which did not extend to full hyphae in comparison to wild type cultures (Fig. 10). This data indicates that *DFG5* and *DCW1* play critical roles in hypha development. Interestingly, this pseudohyphae phenotype was observed for *hog1*Δ** mutant as well (Fig. 10). Although, *hog1*Δ** mutant was not found to be defective in hyphae formation as confirmed previously (Alonso-Monge et al., 1999), its short term (4 h) response to serum was similar to the *DFG5* and *DCW1* mutants.

## Discussion

Fungi are eukaryotes and have a significant structural similarity to human cells. Due to this reason targeting the fungal cell has been relatively difficult as it results in toxicity to host cells. Several antifungal drugs, that are currently available to treat mucosal and disseminated candidiasis, cause adverse toxicity in human patients. The main reason for high morbidity and significant hospital associated costs of treating systemic candidiasis is the rapid development of antifungal drug resistance among *Candida* species (Bondaryk, Kurzatkowski & Staniszewska, 2013; Fothergill et al., 2014). Thus there is an urgent need for novel antifungal drugs and therapeutics which can overcome the frustrating problem of antifungal drug resistance.

The only organelle that is unique to the fungal cell as compared to the human host is the cell wall. The cell wall has several critical functions. It deals with environmental stresses like changes in osmotic pressure, pH and temperature to protect cell integrity (Chaffin, 2008; Free, 2013). It is now understood that several cell wall associated proteins play a role in cell signaling pathways in response to stress. A transcriptional upregulation of genes involved in maintaining cell wall integrity occurs in response to signal transduction (Chaffin, 2008; Dichtl, Samantaray & Wagener, 2016). MAPK signaling pathways are among the various signaling pathways that regulate cell wall biogenesis and integrity. Most importantly, the cell wall plays a critical role in disease pathogenesis as well as in protecting the pathogen from the host immune system ( Cullen & Edgerton, 2016).

The cell wall is a complex structure made of carbohydrates and cell wall mannoproteins. The carbohydrates form an extracellular matrix in which the mannoproteins are cross-linked. The cell wall proteins play important roles in cell physiology as well as in disease pathogenesis. The extracellular matrix is also needed for biofilm formation. Biofilm formation is an important virulence factor for pathogenic fungi in causing local and systemic disease (Costa-Orlandi et al., 2017). In *C. albicans*, various genes are involved in adhesion, extracellular matrix formation, quorum sensing and morphogenesis of biofilms (Fox & Nobile, 2012; Finkel & Mitchell, 2011). Moreover, in *C. albicans* the yeast and hyphae forms have been found to have unique roles in biofilm formation (Finkel & Mitchell, 2011).

The cell wall proteins, Dfg5p and Dcw1p, are predicted mannosidases/glycosyl hydrolases (*gh-76* family). They have been implicated in the cross-linking of cell wall proteins in the cell wall. There are three known ways of cross-linking cell wall proteins in the cell wall matrix of *C. albicans*: (1) by a possible Dfg5p/Dcw1p-mediated cross-linking of N-linked outer chain mannan to the cell wall glucans described in this application, (2) by cross-linking the GPI anchor to the cell wall glucans through an alkaline-sensitive linkage, and (3) PIR (proteins with internal repeats) can be cross-linked into the cell walls by a linkage between a glutamine residue in the PIR repeat and β-1,3-glucan (Free, 2013; Xie & Lipke, 2010). This redundancy in cell wall protein cross-linking is thought to help ensure the formation of a functional cell wall and maintain its integrity. However, the exact enzymatic mechanisms of Dfg5p and Dcw1p have not been demonstrated experimentally.

In this study we examined the functions of Dfg5p and Dcw1p in biofilm formation and Hog1 MAPK signaling by using heterologous mutant strains and conditional mutant strains (Table 1). Using the above *C. albicans* mutant strains we have previously confirmed that the Dfg5p and Dcw1p mannanases (cross-linking enzymes) function in cell wall biogenesis in *C. albicans* (Ao et al., 2015). We have also showed previously that the *DFG5/DCW1* conditional mutants have a cell separation growth phenotype. The mutants are hypersensitive to cell wall stress reagents and to treatment with lyticase (β-glucanase), indicating that the mutant cell walls are weaker than wild type cell walls (Ao et al., 2015). The *DFG5* and *DCW1* mutants produced cell walls containing reduced levels of cell wall proteins and released cell wall proteins into the growth medium. A carbohydrate analysis of the *DFG5/DCW1* mutant cell walls showed that the mannose levels were significantly reduced, indicating a reduced incorporation of cell wall proteins in the wall (Ao et al., 2015). These characteristics are similar to our observations of the *Neurospora crassa DFG5*Δ*/dcw1*Δ** mutants, and demonstrate that *DFG5* and *DCW1* in *C. albicans* function in cross-linking cell wall proteins into the cell wall. However, the substrates of these enzymes and their exact mechanisms of cross-linking cell wall proteins into the cell wall are yet to be determined. But structural studies of the α-mannanases (GH-76 enzymes), have revealed that these enzymes are mainly involved in catalysis and could be potential targets for anti-fungal drug development (Thompson et al., 2015).

We hypothesized that the lethality of the *dfg5*Δ*/dcw1*Δ** mutants in *S. cerevisiae* and *C. albicans* may be a manifestation of additional roles that Dfg5p and Dcw1p play in signal transduction pathways. This has been demonstrated especially in *S. cerevisiae*. Recent studies have shown that the Hog1 and Slt2 signaling pathways are activated in the *dfg5*Δ mutant of *S. cerevisiae* (Nasution et al., 2015). It was also demonstrated in the same study that the expression of genes related to these signaling pathways was altered in the *dfg5*Δ mutant of *S. cerevisiae*, by using RNA sequencing analysis (Nasution et al., 2015). Our preliminary studies have focused on *C. albicans DFG5* and *DCW1* functions at pH 7 which is commonly present in the oral cavity. Also ES195 mutant has defective growth patterns under various cell wall stress conditions but exhibit elevated heat tolerance, which is concurrent with studies in *S. cerevisiae* (Fig. 2). The defect in the ability to overcome cell wall stress could be the result of a weakened cell wall. However, growth of the mutants was not affected by osmotic stress (1 M Sorbitol) (Figs. 3 and 4). Phenotypic analysis of the *DFG5* and *DCW1* mutants indicated a defect in cell separation which was similar to *hog1*Δ** mutant under non-stress conditions (Fig. 5). However, there were minor differences in phenotypes of the mutants with and without osmotic stress, indicating that the *DFG5* and *DCW1* mutations are not indispensable for osmotic stress. We further investigated whether Hog1 MAPK levels were altered in the mutants and found that the basal Hog1 MAPK levels were either reduced or absent in the *DFG5* and *DCW1* mutants (Fig. 6A). However, upon 0.4 M NaCl stress, Hog1 MAPK was induced in the *DFG5* and *DCW1* mutants (Fig. 6B). We further investigated if Hog1 phosphorylation is being affected in these mutants under osmotic stress and found that p-hog1 was slightly reduced in the mutants but not significantly (Fig. 6B) confirming that the *DFG5* and *DCW1* mutations may not affect osmotic stress response via HOG pathway. Taken together, this data indicates that *DFG5* and *DCW1* are not required for HOG dependent osmotic stress response. However, by affecting basal Hog1 MAPK levels, *DFG5* and *DCW1* may possibly regulate other important functions of Hog1.

**Figure 11:**
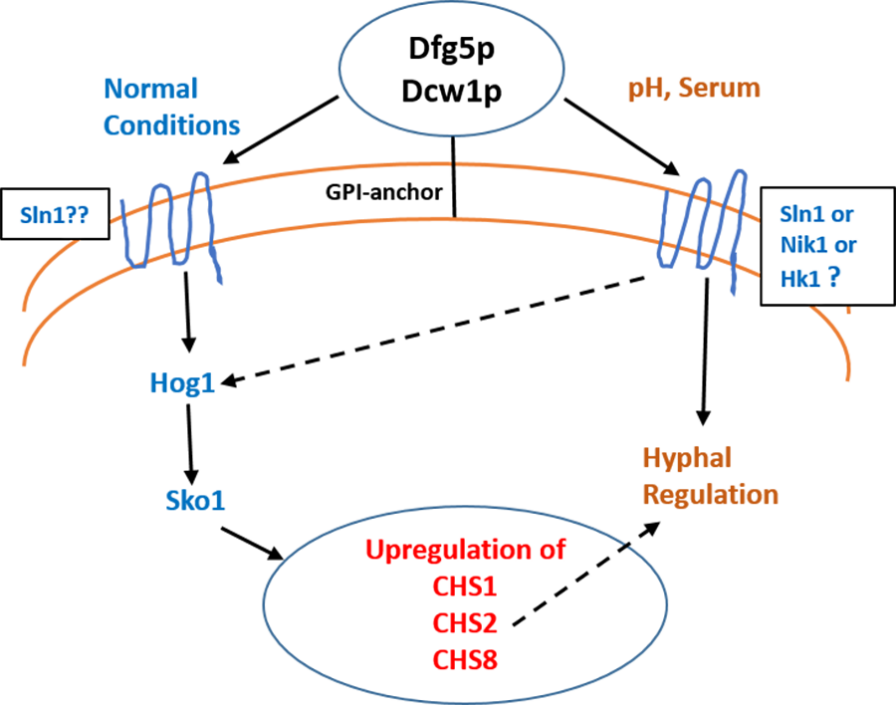
Dfg5p and Dcw1 functional role in regulating hyphal morphogenesis and HOG MAPK pathway. The ligands and downstream components involved in *DFG5/DCW1* dependent hyphal regulation and Hog1 signaling as well as the cell wall protein substrates for *Dfg5p* and *Dcw1p* are to be determined.

Interestingly the HOG pathway under normal or non-stress conditions plays a critical role in chitin synthesis (Munro et al., 2007). *C. albicans* has four genes encoding chitin synthases—*CHS1*, *CHS2*, *CHS3* and *CHS8* with redundant functions in order to ensure cell wall fitness. Of these *CHS1*, *CHS3* and *CHS8* have ATF/CREB elements for Sko1 transcription factor (Munro et al., 2007). Studies in both *S. cerevisiae* and *C. albicans* have shown that hog1 MAPK regulates sko1 transcription factor (Proft et al., 2005; Munro et al., 2007). It was also found that in the *hog1*Δ** mutant, *CHS1*, *CHS2* and *CHS8* transcription was downregulated and *CHS3* was upregulated (Munro et al., 2007). Calcoflour white is a cell wall stress agent that inhibits chitin synthesis and thus causes a compensatory increase in transcriptional regulation of chitin synthases. In this study we show that the ES1 and ES195 conditional mutant are unable to recover by 48 h in the presence of Calcoflour white as compared to wild type (Fig. 2). This could be due to the defect in Hog1 basal levels in the *DFG5* and *DCW1* mutants. This indicates that *DFG5* and *DCW1* mutations may affect chitin synthesis via HOG MAPK pathway. This could further lead to a weakened cell wall and increased susceptibility to cell wall perturbing agents.

Biofilm formation was found to be affected with reduced mass and abnormal phenotypes including reduced hyphal morphogenesis in the *DFG5* and *DCW1* mutants as compared to control strains (Figs. 8 and 9). The phenotype of the ES195+Met/Cys conditional mutant was striking with enlarged cells and pseudohyphae formation (Fig. 9). This phenotype possibly results from a delay in cell cycle progression as *C*. *albicans* pseudohyphal cells spend more time growing in a polarized manner and remain in G_2_ longer than yeast cells (Berman, 2006). Also, pseudohyphal cells do not separate at cytokinesis (Berman, 2006). Additionally, a defect in Hog1 MAPK mediated chitin synthesis may also have a role in the cell separation defect. In a past study, it was demonstrated that treating *hog1*Δ** mutant with commercially available chitinases resulted in the reduction of cell separation (Alonso-Monge et al., 1999). Also, Hog1 is required for the basal transcription of *CHS1*, the chitin synthase that plays a pivotal role in primary septum formation (Munro et al., 2003). Additionally, the chitin synthase *CHS8* is essential for synthesis of long chain chitin microfibrils which are essential for septum formation in yeast and hyphae and may thus play a critical role in hypha morphogenesis (Lenardon et al., 2007). Furthermore, *CHS2* and *CHS8* are required for maintaining cell integrity during early polarized growth in yeast and hyphal cells (Preechasuth et al., 2015). Taken together, it appears that Dfg5p and Dcw1p may affect cell separation and hyphal morphogenesis indirectly via HOG MAPK pathway by regulating chitin synthesis (Fig. 11). This defect in hyphal morphogenesis may further affect biofilm formation. As for the potential upstream elements, Sln1, Nik1 or Hk1 have been shown to be independent of Hog1 under osmotic stress (Yamada-Okabe et al., 1999; Román, Nombela & Pla, 2005). Yet the mutants of these histidine kinases have a defect in hyphal morphogenesis. Thus, Sln1, Nik1 and Hk1 may be involved in regulating Hog1 MAPK under non-stress conditions or in response to serum, pH etc as the mutants of these histidine kinases have defects in hyphal morphogenesis (Yamada-Okabe et al., 1999).

## Conclusions

We conclude that Dfg5p and Dcw1p have distinct functions. While Dfg5p is important for hyphal morphogenesis, Dcw1p could be important for maintaining cell wall integrity. Additionally, these cell wall mannosidases may function in biofilm formation via hyphal regulation and also activate the HOG MAPK pathway to possibly regulate chitin synthesis as depicted in our working model (Fig. 11). As Hog1 is thought to be downstream of Sln1, Nik1 and Hk1, the Dfg5p and Dcw1p cell wall mannosidases could be interacting with these upstream elements. This interaction may involve enzymatic modifications of glycosylation on the osmosensing histidine kinases like Sln1, Nik1 and Hk1 resulting in signal transduction. Located in the cell wall and having enzymatic functions, Dfg5p and Dcw1p are readily accessible to therapeutic agents and could be ideal targets for novel antifungal drugs. Thus, it is not only necessary but novel to further investigate the signaling functions of these GPI-anchored cell wall enzymes. Our future studies will focus on the enzymatic mechanisms of these mannosidases.

##  Supplemental Information

10.7717/peerj.5685/supp-1 Data S1 Raw data for sorbitol growth assay

10.7717/peerj.5685/supp-2 Data S2 Raw data for Fig. 6 Hog1 blot - no stress control.

10.7717/peerj.5685/supp-3 Data S3 Raw data 1 The tables for the numeric data from Figs. 1, 2 and 3, 6B and 7B.

## Additional Information and Declarations

### Data Availability

The following information was supplied regarding data availability: The raw data are included in the Supplemental Files.
