# Supplementary material for: Functions of Candida albicans cell wall glycosidases Dfg5p and Dcw1p in biofilm formation and HOG MAPK pathway
Source: PeerJ. 2018 Sep 28;6:e5685. doi: 10.7717/peerj.5685 (PMC6166624; doi:10.7717/peerj.5685)
Supplement: Data S1 [file peerj-06-5685-s001.pdf]

Plain YNB ("Control")

WT

Time point (h)	Repeat 1	Repeat 2	Repeat 3	AVG OD_{600}	STDEV
0	0.1373	0.1312	0.1176	0.1287	0.010085
2	0.1586	0.1562	0.1384	0.151067	0.011035
4	0.3323	0.3317	0.2937	0.319233	0.022115
6	0.7954	0.7742	0.7366	0.768733	0.029779
8	1.6492	1.6422	1.559	1.6168	0.050178
12	2.5098	2.5114	2.4776	2.4996	0.019069
24	5.377	5.587	5.505	5.489667	0.105836

Time point (h)	Repeat 1
0	0.0683
2	0.0712
4	0.1267
6	0.2562
8	0.5158
12	1.2058
24	3.465

BWP17

Time point (h)	Repeat 1	Repeat 2	Repeat 3	AVG OD_{600}	STDEV
0	0.1102	0.1153	0.1102	0.1119	0.002944
2	0.1553	0.156	0.1539	0.155067	0.001069
4	0.3572	0.3531	0.3552	0.355167	0.00205
6	0.807	0.796	0.8015	0.8015	0.0055
8	1.8508	1.8124	1.8254	1.829533	0.019531
12	3.871	3.871	3.881	3.874333	0.005774
24	5.241	5.173	5.164	5.192667	0.042099

Time point (h)	Repeat 1
0	0.0823
2	0.074
4	0.111
6	0.2114
8	0.4334
12	1.1653
24	3.465

DAY185

Time point (h)	Repeat 1	Repeat 2	Repeat 3	AVG OD_{600}	STDEV
0	0.0942	0.0878	0.0897	0.090567	0.003287
2	0.0969	0.0854	0.085	0.0891	0.006758
4	0.5756	0.5407	0.5243	0.546867	0.0262
6	0.7793	0.7808	0.7869	0.782333	0.004025
8	1.2392	1.1926	1.1912	1.207667	0.027318
12	2.4624	2.4744	2.4564	2.4644	0.009165
24	5.742	5.878	5.782	5.800667	0.069895

Time point (h)	Repeat 1
0	0.0784
2	0.0724
4	0.124
6	0.2248
8	0.4439
12	1.1441
24	4.334

ES1

Time point (h)	Repeat 1	Repeat 2	Repeat 3	AVG OD_{600}	STDEV
0	0.0828	0.0826	0.0828	0.082733	0.000115
2	0.1006	0.0968	0.094	0.097133	0.003313
4	0.205	0.1968	0.1963	0.199367	0.004885
6	0.4772	0.4787	0.442	0.465967	0.020769
8	0.9228	0.9062	0.9048	0.911267	0.010013
12	1.8142	1.7908	1.7832	1.796067	0.016157
24	3.718	3.725	3.853	3.765333	0.076002

Time point (h)	Repeat 1
0	0.0782
2	0.0774
4	0.1534
6	0.2951
8	0.493
12	1.0788
24	2.662

ES195

Time point (h)	Repeat 1	Repeat 2	Repeat 3	AVG OD_{600}	STDEV
0	0.1294	0.1295	0.1295	0.129467	5.77E-05
2	0.1214	0.1173	0.1169	0.118533	0.002491
4	0.2436	0.2393	0.2208	0.234567	0.012115

Time point (h)	Repeat 1
0	0.0748
2	0.0811
4	0.1463

**6**	0.5761	0.5548	0.55	0.5603	0.013892
**8**	1.254	1.2254	1.2	1.226467	0.027016
**12**	2.3034	2.255		2.2792	0.034224
**24**	5.821	5.818	5.756	5.798333	0.036692

**6**	0.2559
**8**	0.4856
**12**	1.0945
**24**	4.348

ES195 +Met/Cys					
**Time point (h)**	**Repeat 1**	**Repeat 2**	**Repeat 3**	**AVG OD_{600}**	**STDEV**
**0**	0.1476	0.1754	0.15	0.157667	0.015404
**2**	0.2936	0.2872	0.3073	0.296033	0.010269
**4**	0.4279	0.4236	0.4309	0.427467	0.003669
**6**	0.528	0.5424	0.5049	0.5251	0.018917
**8**	0.6728	0.6742	0.6644	0.670467	0.0053
**12**	0.7777	0.7964	0.7708	0.781633	0.013246
**24**	0.98	0.9927	0.9619	0.9782	0.015479

**Time point (h)**	**Repeat 1**
**0**	0.1048
**2**	0.1727
**4**	0.2542
**6**	0.3658
**8**	0.4416
**12**	0.5706
**24**	0.6167

YNB + 1M Sorbitol

WT

Repeat 2	Repeat 3	AVG OD_{600}	STDEV
0.0718	0.0726	0.0709	0.002287
0.0742	0.0758	0.073733	0.002335
0.1273	0.1354	0.1298	0.004859
0.2671	0.2592	0.260833	0.005631
0.5086	0.5315	0.518633	0.01171
1.2072	1.2049	1.205967	0.001159
3.61	3.757	3.610667	0.146001

BWP17

Repeat 2	Repeat 3	AVG OD_{600}	STDEV
0.0765	0.0763	0.078367	0.003408
0.07	0.0681	0.0707	0.003012
0.1115	0.1115	0.111333	0.000289
0.212	0.2023	0.208567	0.005435
0.4356	0.4414	0.4368	0.004133
1.1636	1.49	1.272967	0.187958
3.759	3.891	3.705	0.218073

DAY185

Repeat 2	Repeat 3	AVG OD_{600}	STDEV
0.0815	0.0769	0.078933	0.002346
0.0756	0.0822	0.076733	0.004997
0.1271	0.1244	0.125167	0.001686
0.2318	0.2342	0.230267	0.004884
0.462	0.4611	0.455667	0.0102
1.1467	1.1605	1.150433	0.008814
4.438	4.3	4.357333	0.071898

ES1

Repeat 2	Repeat 3	AVG OD_{600}	STDEV
0.0785	0.074	0.0769	0.002516
0.0805	0.0811	0.079667	0.001986
0.158	0.1517	0.154367	0.003259
0.2998	0.2886	0.2945	0.005624
0.5055	0.5061	0.501533	0.007396
1.0412	1.0648	1.0616	0.019003
2.628	2.581	2.623667	0.040673

ES195

Repeat 2	Repeat 3	AVG OD_{600}	STDEV
0.0748	0.0746	0.074733	0.000115
0.0823	0.0853	0.0829	0.002163
0.1429	0.1479	0.1457	0.002553

t-Test: Two-Sample Assuming Equal Variances

WT 0h	*Plain*	*+Sorbitol*
Mean	0.1287	0.0709
Variance	0.00010171	5.23E-06
Observations	3	3
Pooled Variance	5.347E-05	
Hypothesized Mear	0	
df	4	
t Stat	9.68095889	
P(T<=t) one-tail	0.000318544	
t Critical one-tail	2.131846786	
P(T<=t) two-tail	0.000637088	
t Critical two-tail	2.776445105	

t-Test: Two-Sample Assuming Equal Variances

BWP17 0h	*Plain*	*Sorbitol*
Mean	0.1119	0.078367
Variance	8.67E-06	1.16E-05
Observations	3	3
Pooled Variance	1.01417E-05	
Hypothesized Mear	0	
df	4	
t Stat	12.89637608	
P(T<=t) one-tail	0.000104241	
t Critical one-tail	2.131846786	
P(T<=t) two-tail	0.000208483	
t Critical two-tail	2.776445105	

t-Test: Two-Sample Assuming Equal Variances

DAY185 0h	*Plain*	*Sorbitol*
Mean	0.090566667	0.078933
Variance	1.08033E-05	5.5E-06
Observations	3	3
Pooled Variance	8.15333E-06	
Hypothesized Mear	0	
df	4	
t Stat	4.989789247	
P(T<=t) one-tail	0.003772391	
t Critical one-tail	2.131846786	
P(T<=t) two-tail	0.007544782	
t Critical two-tail	2.776445105	

t-Test: Two-Sample Assuming Equal Variances

0.2656	0.2713	0.264267	0.007786
0.4906	0.479	0.485067	0.005818
1.0167	1.1174	1.0762	0.052785
4.28	4.293	4.307	0.036097

ES195 +Met/Cys

Repeat 2	Repeat 3	AVG OD_{600}	STDEV
0.0954	0.109	0.103067	0.006964
0.1316	0.1469	0.1504	0.020772
0.2068	0.2201	0.227033	0.024449
0.3168	0.3325	0.338367	0.025021
0.4007	0.4125	0.418267	0.021051
0.5344	0.5457	0.550233	0.018521
0.6951	0.6253	0.6457	0.042997

ES1 0h	*Plain*	*Sorbitol*
Mean	0.082733333	0.0769
Variance	1.33333E-08	6.33E-06
Observations	3	3
Pooled Variance	3.17167E-06	
Hypothesized Mear	0	
df	4	
t Stat	4.011609532	
P(T<=t) one-tail	0.007987616	
t Critical one-tail	2.131846786	
P(T<=t) two-tail	0.015975232	
t Critical two-tail	2.776445105	

t-Test: Two-Sample Assuming Equal Variances

ES195 0h	*Plain*	*Sorbitol*
Mean	0.129466667	0.074733
Variance	3.33333E-09	1.33E-08
Observations	3	3
Pooled Variance	8.33333E-09	
Hypothesized Mear	0	
df	4	
t Stat	734.3247238	
P(T<=t) one-tail	1.03172E-11	
t Critical one-tail	2.131846786	
P(T<=t) two-tail	2.06345E-11	
t Critical two-tail	2.776445105	

t-Test: Two-Sample Assuming Equal Variances

ES195+M/C 0h	*Plain*	*Sorbitol*
Mean	0.157666667	0.103067
Variance	0.000237293	4.85E-05
Observations	3	3
Pooled Variance	0.000142893	
Hypothesized Mear	0	
df	4	
t Stat	5.594126574	
P(T<=t) one-tail	0.002505573	
t Critical one-tail	2.131846786	
P(T<=t) two-tail	0.005011147	
t Critical two-tail	2.776445105	

t-Test: Two-Sample Assuming Equal Variances

WT 2h	Plain	+Sorbitol
Mean	0.151066667	0.073733
Variance	0.000121773	5.45E-06
Observations	3	3
Pooled Variance	6.36133E-05	
Hypothesized Mear	0	
df	4	
t Stat	11.87512755	
P(T<=t) one-tail	0.000143983	
t Critical one-tail	2.131846786	
P(T<=t) two-tail	0.000287966	
t Critical two-tail	2.776445105	

t-Test: Two-Sample Assuming Equal

WT 4h	Plain
Mean	0.319233333
Variance	0.000489053
Observations	3
Pooled Variance	0.000256332
Hypothesized Mear	0
df	4
t Stat	14.49108488
P(T<=t) one-tail	6.59256E-05
t Critical one-tail	2.131846786
P(T<=t) two-tail	0.000131851
t Critical two-tail	2.776445105

t-Test: Two-Sample Assuming Equal Variances

BWP17 2h	Plain	Sorbitol
Mean	0.155066667	0.0707
Variance	1.14333E-06	9.07E-06
Observations	3	3
Pooled Variance	5.10667E-06	
Hypothesized Mear	0	
df	4	
t Stat	45.72437361	
P(T<=t) one-tail	6.84143E-07	
t Critical one-tail	2.131846786	
P(T<=t) two-tail	1.36829E-06	
t Critical two-tail	2.776445105	

t-Test: Two-Sample Assuming Equal

BWP17 4h	Plain
Mean	0.355166667
Variance	4.20333E-06
Observations	3
Pooled Variance	2.14333E-06
Hypothesized Mear	0
df	4
t Stat	203.9829396
P(T<=t) one-tail	1.73251E-09
t Critical one-tail	2.131846786
P(T<=t) two-tail	3.46502E-09
t Critical two-tail	2.776445105

t-Test: Two-Sample Assuming Equal Variances

DAY185 2h	Plain	Sorbitol
Mean	0.0891	0.076733
Variance	0.00004567	2.5E-05
Observations	3	3
Pooled Variance	3.53217E-05	
Hypothesized Mear	0	
df	4	
t Stat	2.548459232	
P(T<=t) one-tail	0.031704481	
t Critical one-tail	2.131846786	
P(T<=t) two-tail	0.063408961	
t Critical two-tail	2.776445105	

t-Test: Two-Sample Assuming Equal

DAY185 4h	Plain
Mean	0.546866667
Variance	0.000686443
Observations	3
Pooled Variance	0.000344643
Hypothesized Mear	0
df	4
t Stat	27.82045891
P(T<=t) one-tail	4.96515E-06
t Critical one-tail	2.131846786
P(T<=t) two-tail	9.9303E-06
t Critical two-tail	2.776445105

t-Test: Two-Sample Assuming Equal Variances

t-Test: Two-Sample Assuming Equal

ES1 2h	Plain	Sorbitol
Mean	0.097133333	0.079667
Variance	1.09733E-05	3.94E-06
Observations	3	3
Pooled Variance	7.45833E-06	
Hypothesized Mear	0	
df	4	
t Stat	7.833119772	
P(T<=t) one-tail	0.000717149	
t Critical one-tail	2.131846786	
P(T<=t) two-tail	0.001434297	
t Critical two-tail	2.776445105	

ES1 4h	Plain
Mean	0.199366667
Variance	2.38633E-05
Observations	3
Pooled Variance	1.72433E-05
Hypothesized Mear	0
df	4
t Stat	13.27234101
P(T<=t) one-tail	9.31261E-05
t Critical one-tail	2.131846786
P(T<=t) two-tail	0.000186252
t Critical two-tail	2.776445105

t-Test: Two-Sample Assuming Equal Variances

t-Test: Two-Sample Assuming Equal

ES195 2h	Plain	Sorbitol
Mean	0.118533333	0.0829
Variance	6.20333E-06	4.68E-06
Observations	3	3
Pooled Variance	5.44167E-06	
Hypothesized Mear	0	
df	4	
t Stat	18.70837698	
P(T<=t) one-tail	2.40298E-05	
t Critical one-tail	2.131846786	
P(T<=t) two-tail	4.80595E-05	
t Critical two-tail	2.776445105	

ES195 4h	Plain
Mean	0.234566667
Variance	0.000146763
Observations	3
Pooled Variance	7.66417E-05
Hypothesized Mear	0
df	4
t Stat	12.43231627
P(T<=t) one-tail	0.00012034
t Critical one-tail	2.131846786
P(T<=t) two-tail	0.00024068
t Critical two-tail	2.776445105

t-Test: Two-Sample Assuming Equal Variances

t-Test: Two-Sample Assuming Equal

ES195+M/C 2h	Plain	Sorbitol
Mean	0.296033333	0.1504
Variance	0.000105443	0.000431
Observations	3	3
Pooled Variance	0.000268467	
Hypothesized Mear	0	
df	4	
t Stat	10.88582214	
P(T<=t) one-tail	0.00020213	
t Critical one-tail	2.131846786	
P(T<=t) two-tail	0.00040426	
t Critical two-tail	2.776445105	

ES195+M/C 4h	Plain
Mean	0.427466667
Variance	1.34633E-05
Observations	3
Pooled Variance	0.000305603
Hypothesized Mear	0
df	4
t Stat	14.04224465
P(T<=t) one-tail	7.46161E-05
t Critical one-tail	2.131846786
P(T<=t) two-tail	0.000149232
t Critical two-tail	2.776445105

Variances
+Sorbitol
0.1298
2.36E-05
3

t-Test: Two-Sample Assuming Equal Variances

WT 6h	Plain	+Sorbitol
Mean	0.768733333	0.260833
Variance	0.000886773	3.17E-05
Observations	3	3
Pooled Variance	0.000459238	
Hypothesized Mear	0	
df	4	
t Stat	29.02717534	
P(T<=t) one-tail	4.19251E-06	
t Critical one-tail	2.131846786	
P(T<=t) two-tail	8.38501E-06	
t Critical two-tail	2.776445105	

t-Test: Two-Sample

WT 8h
Mean
Variance
Observations
Pooled Variance
Hypothesized Mear
df
t Stat
P(T<=t) one-tail
t Critical one-tail
P(T<=t) two-tail
t Critical two-tail

Variances
Sorbitol
0.111333
8.33E-08
3

t-Test: Two-Sample Assuming Equal Variances

BWP17 6h	Plain	Sorbitol
Mean	0.208566667	0.111333
Variance	2.95433E-05	8.33E-08
Observations	3	3
Pooled Variance	1.48133E-05	
Hypothesized Mear	0	
df	4	
t Stat	30.9410043	
P(T<=t) one-tail	3.25061E-06	
t Critical one-tail	2.131846786	
P(T<=t) two-tail	6.50123E-06	
t Critical two-tail	2.776445105	

t-Test: Two-Sample

BWP17 8h
Mean
Variance
Observations
Pooled Variance
Hypothesized Mear
df
t Stat
P(T<=t) one-tail
t Critical one-tail
P(T<=t) two-tail
t Critical two-tail

Variances
Sorbitol
0.125167
2.84E-06
3

t-Test: Two-Sample Assuming Equal Variances

DAY185 6h	Plain	Sorbitol
Mean	0.782333333	0.230267
Variance	1.62033E-05	2.39E-05
Observations	3	3
Pooled Variance	2.00283E-05	
Hypothesized Mear	0	
df	4	
t Stat	151.0827042	
P(T<=t) one-tail	5.75619E-09	
t Critical one-tail	2.131846786	
P(T<=t) two-tail	1.15124E-08	
t Critical two-tail	2.776445105	

t-Test: Two-Sample

DAY185 8h
Mean
Variance
Observations
Pooled Variance
Hypothesized Mear
df
t Stat
P(T<=t) one-tail
t Critical one-tail
P(T<=t) two-tail
t Critical two-tail

Variances

t-Test: Two-Sample Assuming Equal Variances

t-Test: Two-Sample

Sorbitol
0.154367
1.06E-05
3

ES1 6h	Plain	Sorbitol
Mean	0.465966667	0.2945
Variance	0.000431363	3.16E-05
Observations	3	3
Pooled Variance	0.000231497	
Hypothesized Mear	0	
df	4	
t Stat	13.8023477	
P(T<=t) one-tail	7.98477E-05	
t Critical one-tail	2.131846786	
P(T<=t) two-tail	0.000159695	
t Critical two-tail	2.776445105	

ES1 8h
Mean
Variance
Observations
Pooled Variance
Hypothesized Mear
df
t Stat
P(T<=t) one-tail
t Critical one-tail
P(T<=t) two-tail
t Critical two-tail

Variances

t-Test: Two-Sample Assuming Equal Variances

t-Test: Two-Sample

Sorbitol
0.1457
6.52E-06
3

ES195 6h	Plain	Sorbitol
Mean	0.5603	0.264267
Variance	0.00019299	6.06E-05
Observations	3	3
Pooled Variance	0.000126807	
Hypothesized Mear	0	
df	4	
t Stat	32.19698473	
P(T<=t) one-tail	2.77378E-06	
t Critical one-tail	2.131846786	
P(T<=t) two-tail	5.54757E-06	
t Critical two-tail	2.776445105	

ES195 8h
Mean
Variance
Observations
Pooled Variance
Hypothesized Mear
df
t Stat
P(T<=t) one-tail
t Critical one-tail
P(T<=t) two-tail
t Critical two-tail

Variances

t-Test: Two-Sample Assuming Equal Variances

t-Test: Two-Sample

Sorbitol
0.227033
0.000598
3

ES195+M/C 6h	Plain	Sorbitol
Mean	0.5251	0.338367
Variance	0.00035787	0.000626
Observations	3	3
Pooled Variance	0.000491967	
Hypothesized Mear	0	
df	4	
t Stat	10.31097278	
P(T<=t) one-tail	0.000249557	
t Critical one-tail	2.131846786	
P(T<=t) two-tail	0.000499113	
t Critical two-tail	2.776445105	

ES195+M/C 8h
Mean
Variance
Observations
Pooled Variance
Hypothesized Mear
df
t Stat
P(T<=t) one-tail
t Critical one-tail
P(T<=t) two-tail
t Critical two-tail

Assuming Equal Variances

Plain	+Sorbitol
1.6168	0.518633
0.00251788	0.000137
3	3
0.001327502	
0	
4	
36.9144455	
1.60773E-06	
2.131846786	
3.21547E-06	
2.776445105	

t-Test: Two-Sample Assuming Equal Variances

WT 12h	Plain	+Sorbitol
Mean	2.4996	1.205967
Variance	0.00036364	1.34E-06
Observations	3	3
Pooled Variance	0.000182492	
Hypothesized Mear	0	
df	4	
t Stat	117.2830654	
P(T<=t) one-tail	1.58478E-08	
t Critical one-tail	2.131846786	
P(T<=t) two-tail	3.16956E-08	
t Critical two-tail	2.776445105	

Assuming Equal Variances

Plain	Sorbitol
1.829533333	0.4368
0.000381453	1.71E-05
3	3
0.000199267	
0	
4	
120.8359808	
1.40649E-08	
2.131846786	
2.81299E-08	
2.776445105	

t-Test: Two-Sample Assuming Equal Variances

BWP17 12h	Plain	Sorbitol
Mean	3.874333333	1.272967
Variance	3.33333E-05	0.035328
Observations	3	3
Pooled Variance	0.017680828	
Hypothesized Mear	0	
df	4	
t Stat	23.96050112	
P(T<=t) one-tail	8.99728E-06	
t Critical one-tail	2.131846786	
P(T<=t) two-tail	1.79946E-05	
t Critical two-tail	2.776445105	

Assuming Equal Variances

Plain	Sorbitol
1.207666667	0.455667
0.000746253	0.000104
3	3
0.000425148	
0	
4	
44.66766287	
7.51101E-07	
2.131846786	
1.5022E-06	
2.776445105	

t-Test: Two-Sample Assuming Equal Variances

DAY185 12h	Plain	Sorbitol
Mean	2.4644	1.150433
Variance	8.4E-05	7.77E-05
Observations	3	3
Pooled Variance	8.08467E-05	
Hypothesized Mear	0	
df	4	
t Stat	178.9776981	
P(T<=t) one-tail	2.92304E-09	
t Critical one-tail	2.131846786	
P(T<=t) two-tail	5.84609E-09	
t Critical two-tail	2.776445105	

Assuming Equal Variances

t-Test: Two-Sample Assuming Equal Variances

Plain	Sorbitol
0.911266667	0.501533
0.000100253	5.47E-05
3	3
7.74783E-05	
0	
4	
57.01075487	
2.83403E-07	
2.131846786	
5.66805E-07	
2.776445105	

ES1 12h	Plain	Sorbitol
Mean	1.796066667	1.0616
Variance	0.000261053	0.000361
Observations	3	3
Pooled Variance	0.000311087	
Hypothesized Mear	0	
df	4	
t Stat	51.00080384	
P(T<=t) one-tail	4.42284E-07	
t Critical one-tail	2.131846786	
P(T<=t) two-tail	8.84567E-07	
t Critical two-tail	2.776445105	

Assuming Equal Variances

t-Test: Two-Sample Assuming Equal Variances

Plain	Sorbitol
1.226466667	0.418267
0.000729853	0.000443
3	3
0.000586498	
0	
4	
40.87248889	
1.07069E-06	
2.131846786	
2.14139E-06	
2.776445105	

ES195 12h	Plain	Sorbitol
Mean	2.2792	0.418267
Variance	0.00117128	0.000443
Observations	2	3
Pooled Variance	0.000685856	
Hypothesized Mear	0	
df	3	
t Stat	77.84040873	
P(T<=t) one-tail	2.33651E-06	
t Critical one-tail	2.353363435	
P(T<=t) two-tail	4.67302E-06	
t Critical two-tail	3.182446305	

Assuming Equal Variances

t-Test: Two-Sample Assuming Equal Variances

Plain	Sorbitol
0.670466667	0.418267
2.80933E-05	0.000443
3	3
0.000235618	
0	
4	
20.12269627	
1.79995E-05	
2.131846786	
3.5999E-05	
2.776445105	

ES195+M/C 12h	Plain	Sorbitol
Mean	0.781633333	0.550233
Variance	0.000175443	0.000343
Observations	3	3
Pooled Variance	0.000259233	
Hypothesized Mear	0	
df	4	
t Stat	17.60206263	
P(T<=t) one-tail	3.059E-05	
t Critical one-tail	2.131846786	
P(T<=t) two-tail	6.118E-05	
t Critical two-tail	2.776445105	

t-Test: Two-Sample Assuming Equal Variances

WT 24h	Plain	+Sorbitol
Mean	5.489666667	3.610667
Variance	0.011201333	0.021316
Observations	3	3
Pooled Variance	0.016258833	
Hypothesized Mear	0	
df	4	
t Stat	18.04794339	
P(T<=t) one-tail	2.7706E-05	
t Critical one-tail	2.131846786	
P(T<=t) two-tail	5.5412E-05	
t Critical two-tail	2.776445105	

t-Test: Two-Sample Assuming Equal Variances

BWP17 24h	Plain	Sorbitol
Mean	5.192666667	3.705
Variance	0.001772333	0.047556
Observations	3	3
Pooled Variance	0.024664167	
Hypothesized Mear	0	
df	4	
t Stat	11.60160402	
P(T<=t) one-tail	0.000157703	
t Critical one-tail	2.131846786	
P(T<=t) two-tail	0.000315405	
t Critical two-tail	2.776445105	

t-Test: Two-Sample Assuming Equal Variances

DAY185 24h	Plain	Sorbitol
Mean	5.800666667	4.357333
Variance	0.004885333	0.005169
Observations	3	3
Pooled Variance	0.005027333	
Hypothesized Mear	0	
df	4	
t Stat	24.93121422	
P(T<=t) one-tail	7.68252E-06	
t Critical one-tail	2.131846786	
P(T<=t) two-tail	1.5365E-05	
t Critical two-tail	2.776445105	

t-Test: Two-Sample Assuming Equal Variances

ES1 24h	Plain	Sorbitol
Mean	3.765333333	2.623667
Variance	0.005776333	0.001654
Observations	3	3
Pooled Variance	0.003715333	
Hypothesized Mear	0	
df	4	
t Stat	22.93961151	
P(T<=t) one-tail	1.06978E-05	
t Critical one-tail	2.131846786	
P(T<=t) two-tail	2.13956E-05	
t Critical two-tail	2.776445105	

t-Test: Two-Sample Assuming Equal Variances

ES195 24h	Plain	Sorbitol
Mean	5.798333333	4.307
Variance	0.001346333	0.001303
Observations	3	3
Pooled Variance	0.001324667	
Hypothesized Mear	0	
df	4	
t Stat	50.18420522	
P(T<=t) one-tail	4.71742E-07	
t Critical one-tail	2.131846786	
P(T<=t) two-tail	9.43483E-07	
t Critical two-tail	2.776445105	

t-Test: Two-Sample Assuming Equal Variances

ES195+M/C 24h	Plain	Sorbitol
Mean	0.9782	0.6457
Variance	0.00023959	0.001849
Observations	3	3
Pooled Variance	0.001044175	
Hypothesized Mear	0	
df	4	
t Stat	12.60232379	
P(T<=t) one-tail	0.000114105	
t Critical one-tail	2.131846786	
P(T<=t) two-tail	0.00022821	
t Critical two-tail	2.776445105	
