# Supplementary figures and images for: Functions of Candida albicans cell wall glycosidases Dfg5p and Dcw1p in biofilm formation and HOG MAPK pathway
Source: PeerJ. 2018 Sep 28;6:e5685. doi: 10.7717/peerj.5685 (PMC6166624; doi:10.7717/peerj.5685)

# Hog1 Blot – No Stress Control

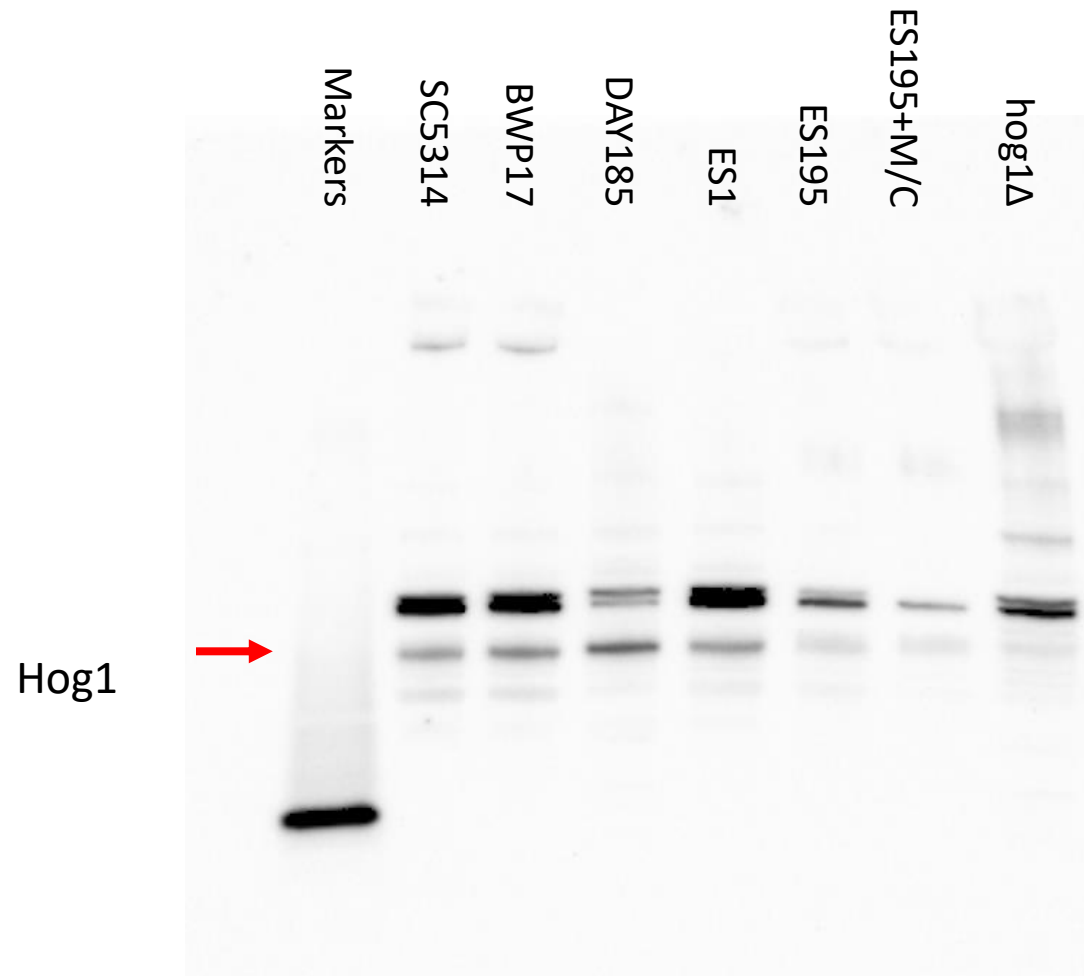

75 sec. ECL image only

# Coomassie Blot

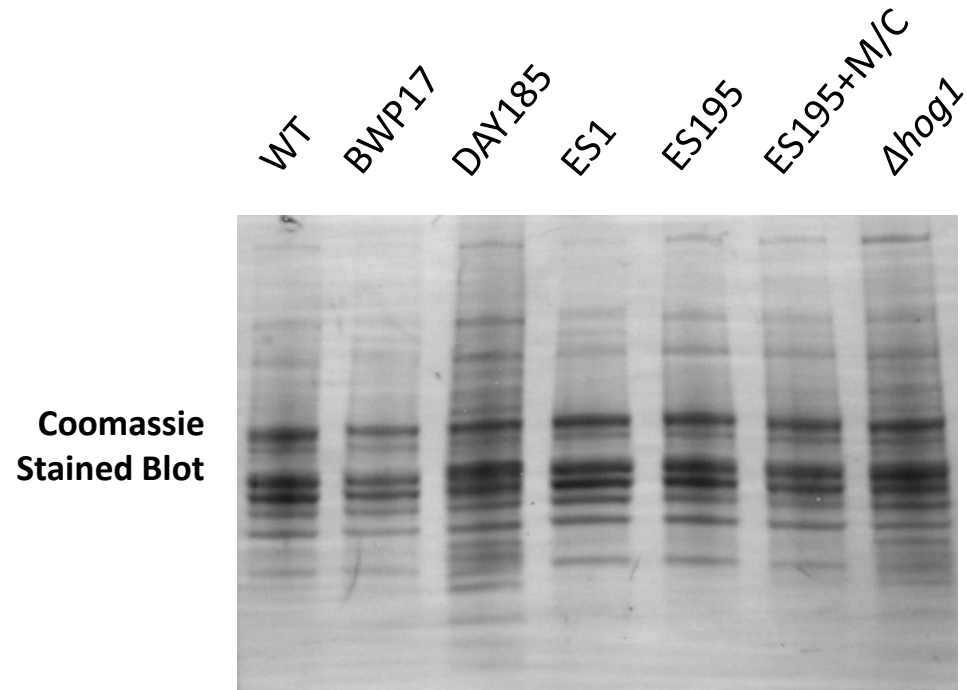

|                |   |   |   |   |   |   |   |
|----------------|---|---|---|---|---|---|---|
| 0.4M NaCl      | - | - | - | - | - | - | - |
| 5mM Methionine | - | - | - | - | - | + | - |
| 2mM Cysteine   | - | - | - | - | - | + | - |

Supplement: Data S2 — Hog1 blot - no stress control. [file peerj-06-5685-s002.pdf]
