# Supplementary material for: Functions of Candida albicans cell wall glycosidases Dfg5p and Dcw1p in biofilm formation and HOG MAPK pathway
Source: PeerJ. 2018 Sep 28;6:e5685. doi: 10.7717/peerj.5685 (PMC6166624; doi:10.7717/peerj.5685)
Supplement: Data S3 — The tables for the numeric data from Figs. 1, 2 and 3, 6B and 7B. [file peerj-06-5685-s003.pdf]

Raw Data for Figure 1: OD600 Readings were obtained for Control and Mutant Strains and used for generating a graph

WT					
Time point (h)	Repeat 1	Repeat 2	Repeat 3	AVG OD_{600}	STDEV
0	0.1373	0.1312	0.1176	0.1287	0.01008514
2	0.1586	0.1562	0.1384	0.1510667	0.0110351
4	0.3323	0.3317	0.2937	0.3192333	0.02211455
6	0.7954	0.7742	0.7366	0.7687333	0.02977874
8	1.6492	1.6422	1.559	1.6168	0.05017848
12	2.5098	2.5114	2.4776	2.4996	0.01906935
24	5.377	5.587	5.505	5.4896667	0.10583635

ES1					
Time point (h)	Repeat 1	Repeat 2	Repeat 3	AVG OD_{600}	STDEV
0	0.0828	0.0826	0.0828	0.0827333	0.00011547
2	0.1006	0.0968	0.094	0.0971333	0.0033126
4	0.205	0.1968	0.1963	0.1993667	0.00488501
6	0.4772	0.4787	0.442	0.4659667	0.02076929
8	0.9228	0.9062	0.9048	0.9112667	0.01001266
12	1.8142	1.7908	1.7832	1.7960667	0.01615714
24	3.718	3.725	3.853	3.7653333	0.07600219

BWP17					
Time point (h)	Repeat 1	Repeat 2	Repeat 3	AVG OD_{600}	STDEV
0	0.1102	0.1153	0.1102	0.1119	0.00294449
2	0.1553	0.156	0.1539	0.1550667	0.00106927
4	0.3572	0.3531	0.3552	0.3551667	0.0020502
6	0.807	0.796	0.8015	0.8015	0.0055
8	1.8508	1.8124	1.8254	1.8295333	0.01953083
12	3.871	3.871	3.881	3.8743333	0.0057735
24	5.241	5.173	5.164	5.1926667	0.04209909

ES195					
Time point (h)	Repeat 1	Repeat 2	Repeat 3	AVG OD_{600}	STDEV
0	0.1294	0.1295	0.1295	0.1294667	5.7735E-05
2	0.1214	0.1173	0.1169	0.1185333	0.00249065
4	0.2436	0.2393	0.2208	0.2345667	0.01211459
6	0.5761	0.5548	0.55	0.5603	0.01389208
8	1.254	1.2254	1.2	1.2264667	0.0270158
12	2.3034	2.255		2.2792	0.03422397
24	5.821	5.818	5.756	5.7983333	0.03669242

DAY185					
Time point (h)	Repeat 1	Repeat 2	Repeat 3	AVG OD_{600}	STDEV
0	0.0942	0.0878	0.0897	0.0905667	0.00328684
2	0.0969	0.0854	0.085	0.0891	0.00675796
4	0.5756	0.5407	0.5243	0.5468667	0.02620006
6	0.7793	0.7808	0.7869	0.7823333	0.00402534
8	1.2392	1.1926	1.1912	1.2076667	0.02731764
12	2.4624	2.4744	2.4564	2.4644	0.00916515
24	5.742	5.878	5.782	5.8006667	0.06989516

ES195 +Met/Cys					
Time point (h)	Repeat 1	Repeat 2	Repeat 3	AVG OD_{600}	STDEV
0	0.1476	0.1754	0.15	0.1576667	0.01540433
2	0.2936	0.2872	0.3073	0.2960333	0.01026856
4	0.4279	0.4236	0.4309	0.4274667	0.00366924
6	0.528	0.5424	0.5049	0.5251	0.01891745
8	0.6728	0.6742	0.6644	0.6704667	0.00530031
12	0.7777	0.7964	0.7708	0.7816333	0.0132455
24	0.98	0.9927	0.9619	0.9782	0.0154787

Raw Data for Figure 8: Biofilm dry mass was determined for Wild Type and Mutant Strains and used for generating a graph

Strain	Repeat #	Tube #	Empty tube(g)	Tube+cells(g)	Dry cell mass(g)	Dry cell mass(mg)	Avg. Dry cell mass (mg)	STDEV
WT (SC5314)	1	1	0.9418	0.9471	0.0053	5.3	4.983333333	0.4579
		2	0.9361	0.9402	0.0041	4.1		
		3	0.9481	0.953	0.0049	4.9		
	2	1	0.9859	0.9911	0.0052	5.2		
		2	0.9365	0.9416	0.0051	5.1		
		3	0.9414	0.9467	0.0053	5.3		
BWP17	1	1	0.9957	1.0008	0.0051	5.1	4.783333333	0.5529
		2	0.9973	1.0018	0.0045	4.5		
		3	0.9424	0.9463	0.0039	3.9		
	2	1	0.9973	1.002	0.0047	4.7		
		2	0.9417	0.9467	0.005	5		
		3	0.9288	0.9343	0.0055	5.5		
DAY185	1	1	0.9438	0.95	0.0062	6.2	6.166666667	0.1862
		2	0.9825	0.9887	0.0062	6.2		
		3	0.9459	0.9521	0.0062	6.2		
	2	1	0.9412	0.9475	0.0063	6.3		
		2	0.9421	0.9479	0.0058	5.8		
		3	0.9844	0.9907	0.0063	6.3		
ES1	1	1	0.9333	0.9361	0.0028	2.8	2.916666667	0.5115
		2	1.0096	1.0126	0.003	3		
		3	0.9284	0.9318	0.0034	3.4		
	2	1	0.9417	0.9452	0.0035	3.5		
		2	0.9641	0.9668	0.0027	2.7		
		3	0.9468	0.9489	0.0021	2.1		
ES195	1	1	1.0209	1.0267	0.0058	5.8	5.433333333	0.3559
		2	0.9823	0.9878	0.0055	5.5		
		3	0.9412	0.9467	0.0055	5.5		
	2	1	0.9337	0.939	0.0053	5.3		
		2	0.9486	0.9543	0.0057	5.7		
		3	0.9292	0.934	0.0048	4.8		
ES195+Met/Cys	1	1	0.9595	0.963	0.0035	3.5	3.583333333	0.2041
		2	0.9429	0.9465	0.0036	3.6		
		3	0.9359	0.9392	0.0033	3.3		
	2	1	0.9992	1.0029	0.0037	3.7		
		2	0.9332	0.9371	0.0039	3.9		
		3	0.948	0.9515	0.0035	3.5		

p values from t-tests

Between		p <
**Group 1**	**Group 2**	
WT	ES1	2.39E-05
WT	ES195	0.087
WT	ES195+M/C	4.51E-05
BWP17	ES1	0.0001
BWP17	ES195	0.04
BWP17	ES195+M/C	0.0005
DAY185	ES1	4.46E-08
DAY185	ES195	0.001
DAY185	ES195+M/C	5.68E-10
ES1	ES195	1.76E-06
ES1	ES195+M/C	0.01
ES195	ES195+M/C	6.35E-07

Raw Data for Figure 9: Biofilm Microscopy Analysis Repeat 1

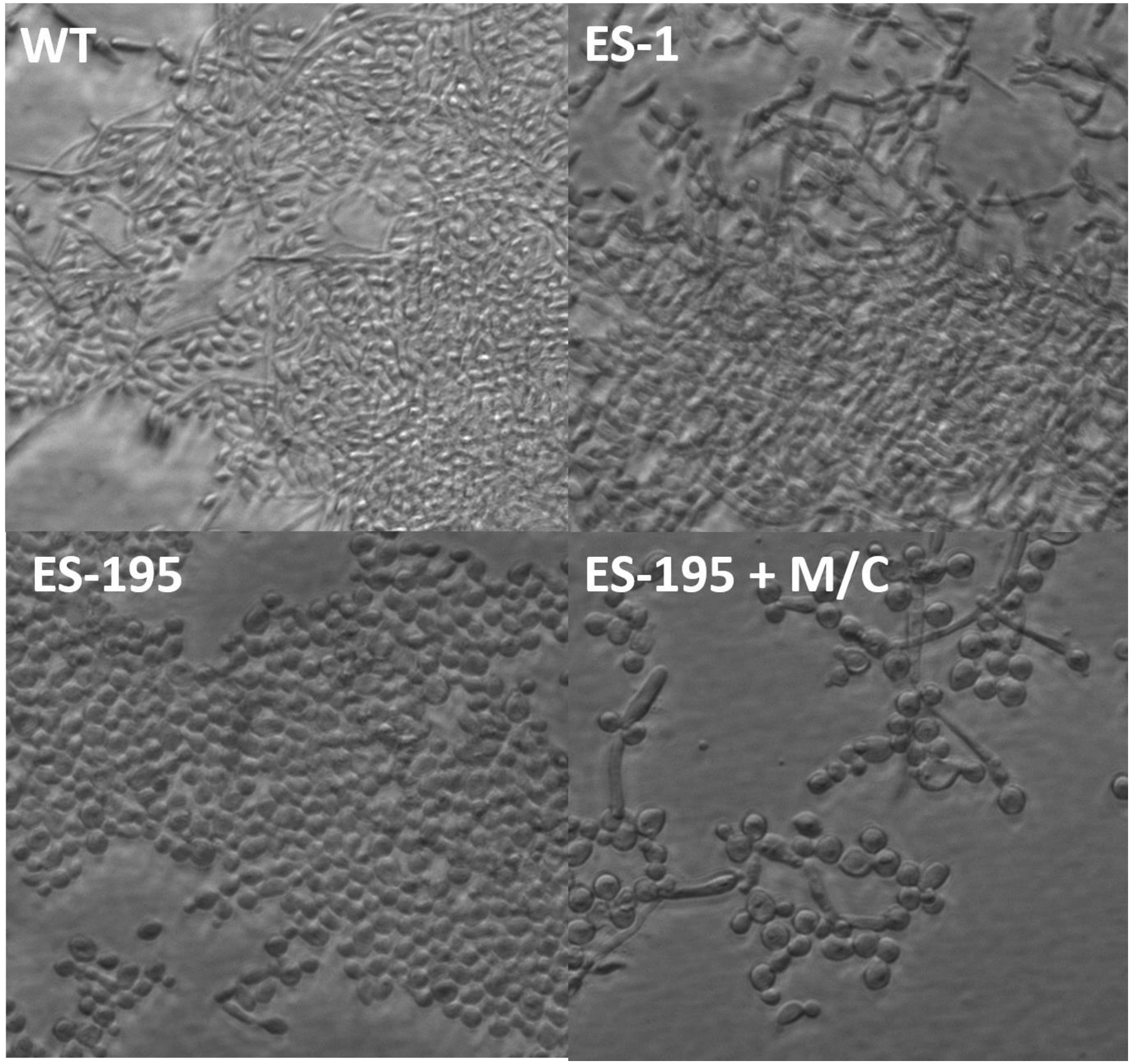

Raw Data for Figure 9: Microscopy Analysis Repeat 2

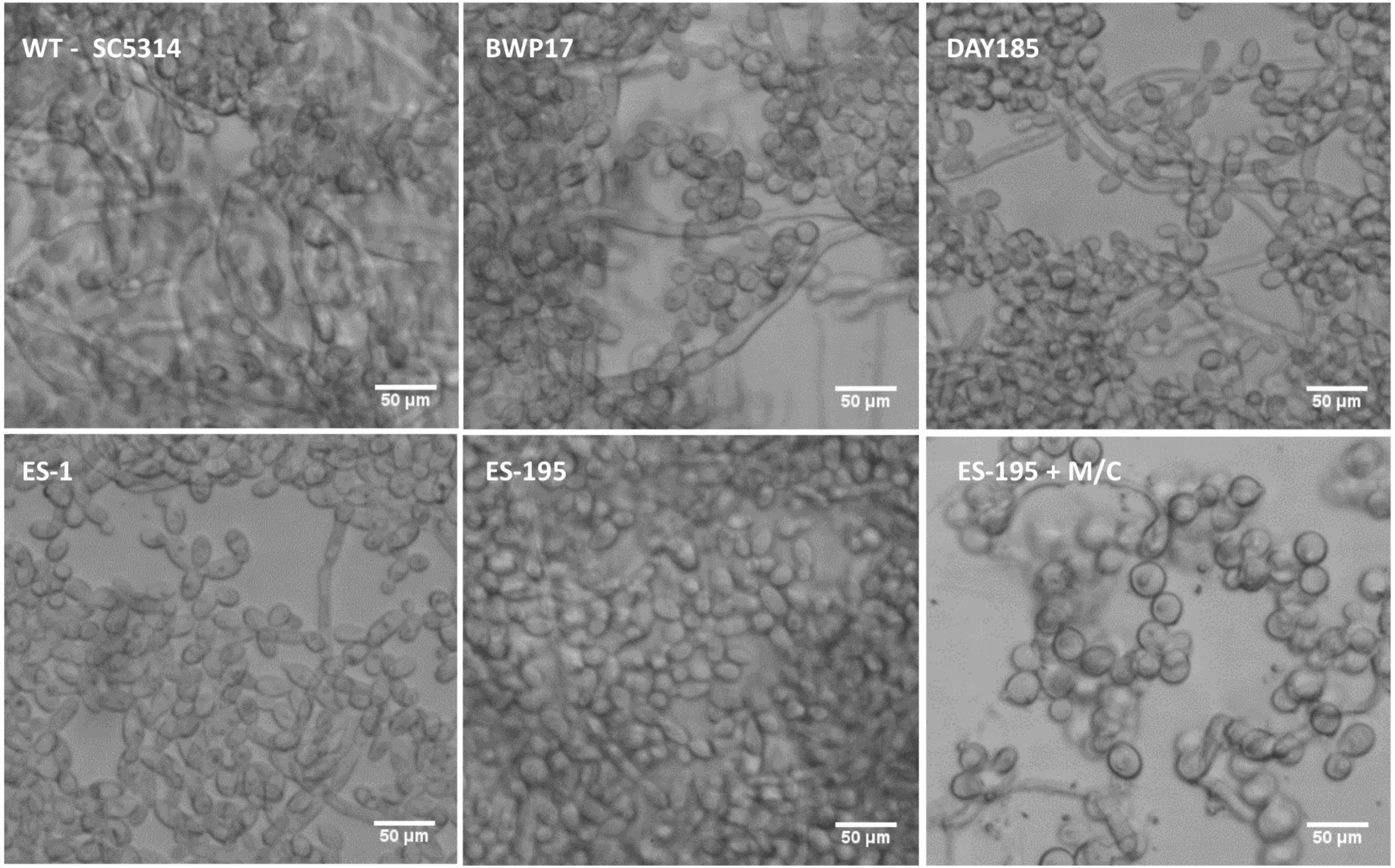

Antibody Information:

Antibody	For	Company	Cat#	Animal		Dilution	Buffer
Phospho P38	P-Hog1	Cell Signaling	9211	Rabbit	Polyclonal	1:10,000	TBST, 5%BSA
Hog1	Total Hog1	Santa Cruz Biotechnology	Y-215	Rabbit	Polyclonal	1:6,666	TBST, 5% Nonfat milk
G6PDH	G6PDH	Sigma	A9521	Rabbit	Polyclonal	1:10,000	TBST, 5% Nonfat milk

- TBST: TBS, 0.05% Tween20
- Primary antibody incubations done overnight

Figure 7:
Total hog1 – 0.4 M NaCl Stress Induced <u>Blot # 1</u>

Total hog1
43 kDa

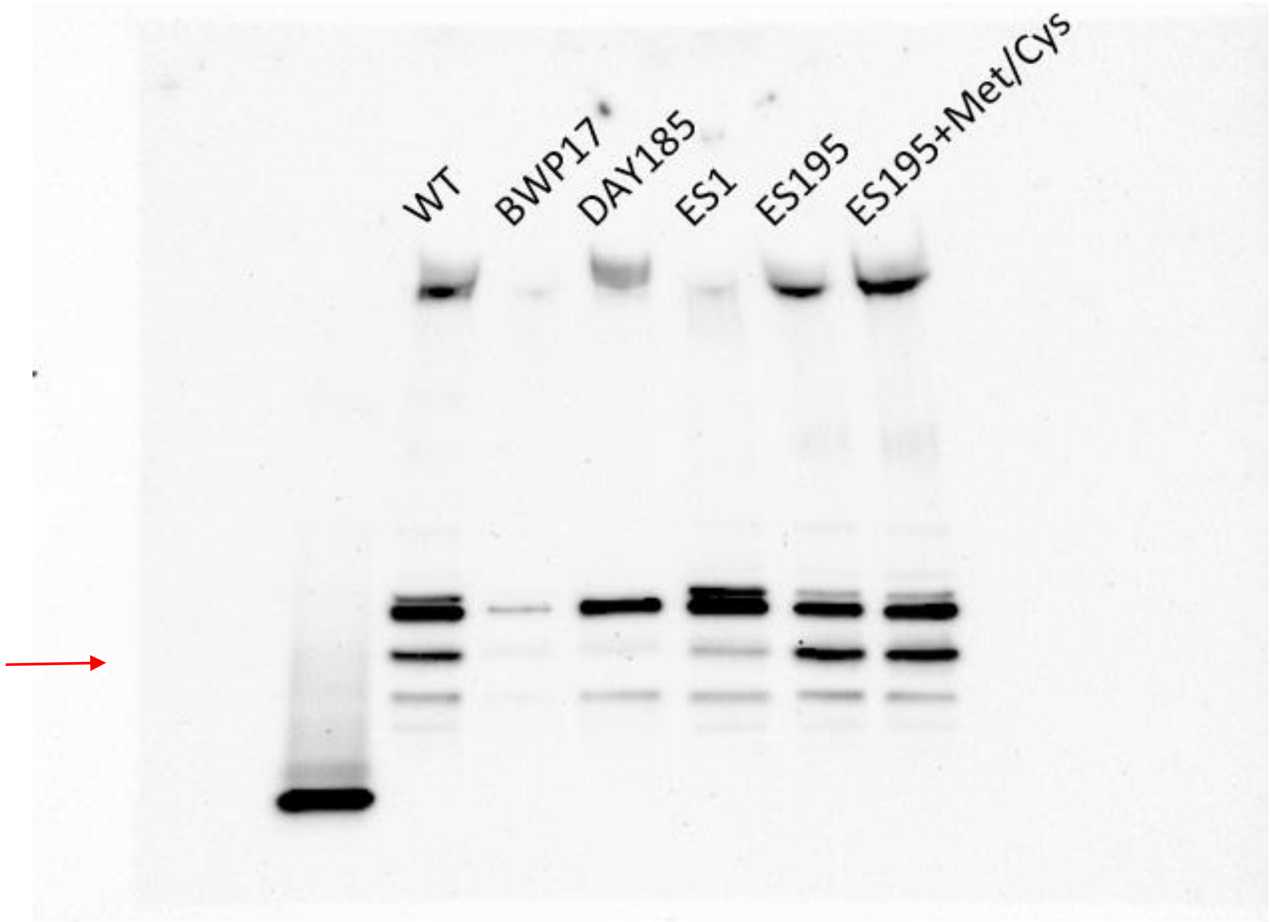

Total-HOG1p

Figure 7: Coomassie stain for HOG1 Blot #1

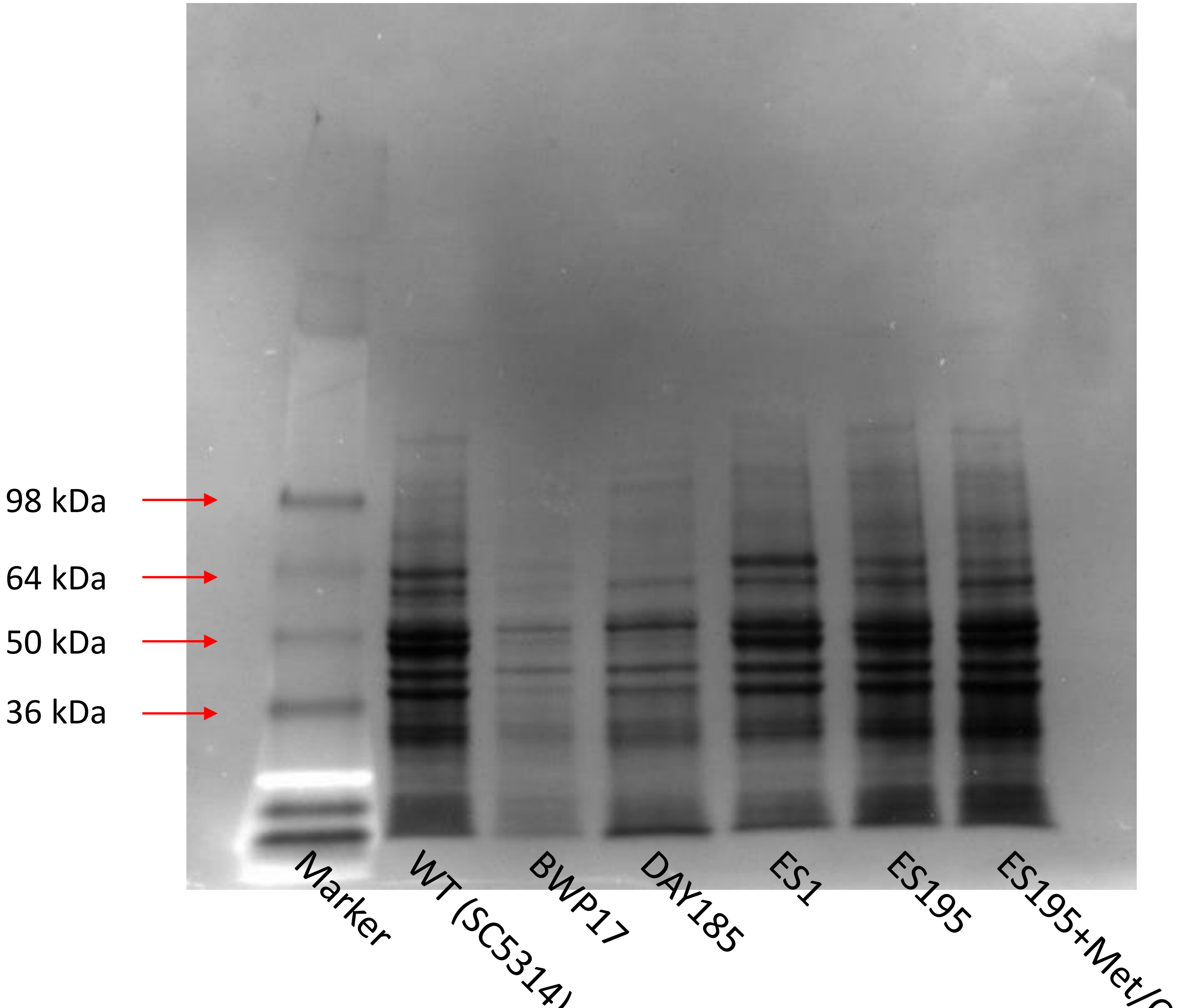

Figure 7:
Total hog1 – 0.4 M NaCl Stress Induced <u>Blot # 2</u>

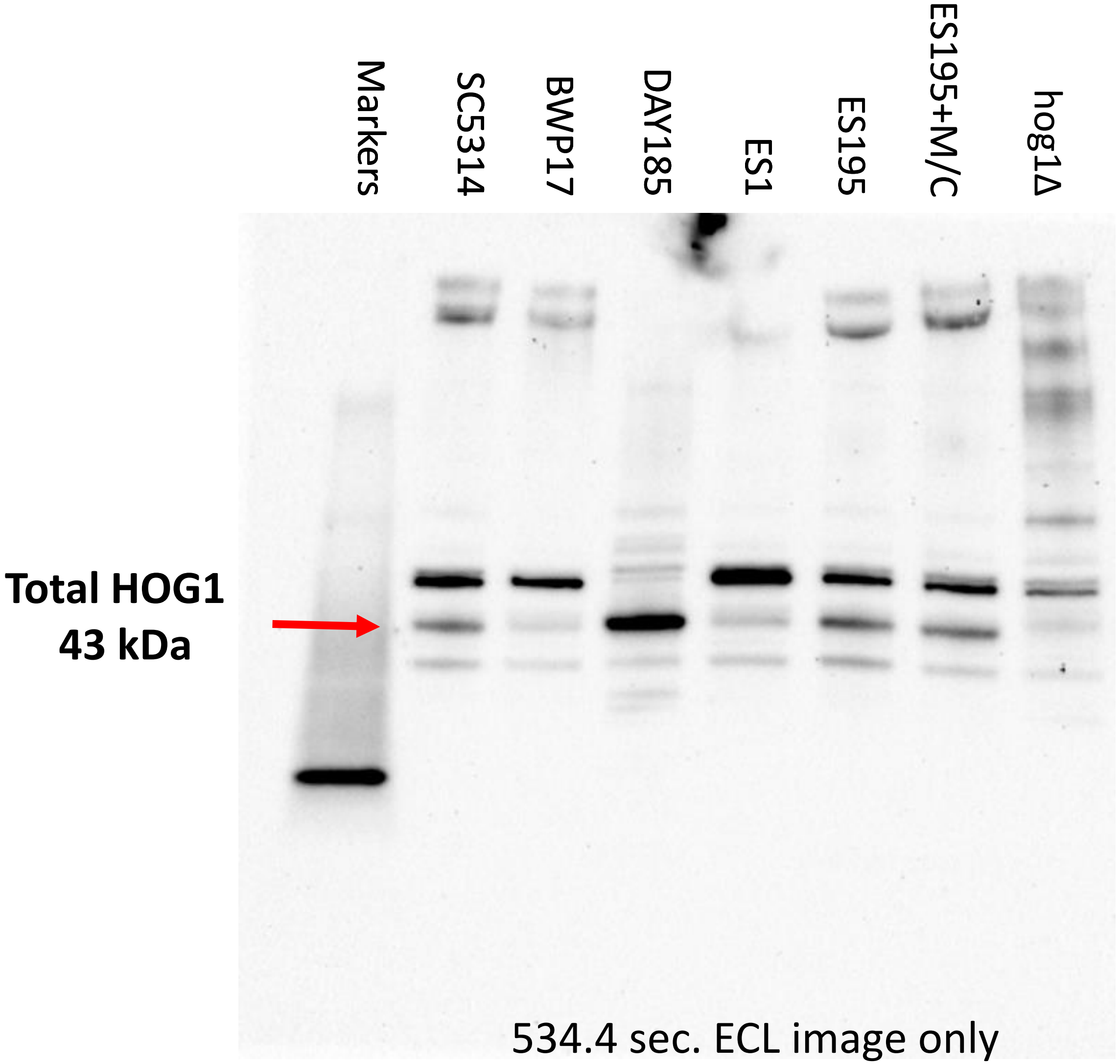

Figure 7: Commassie stain for Blot # 2

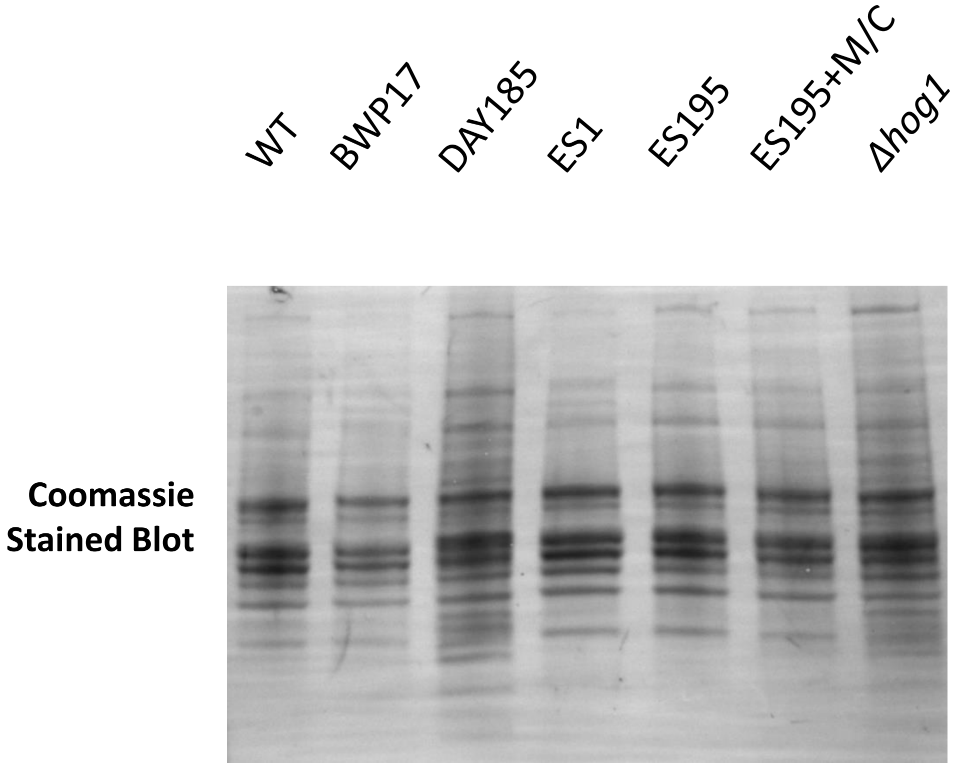

Figure 8 – phospho-Hog1 blot

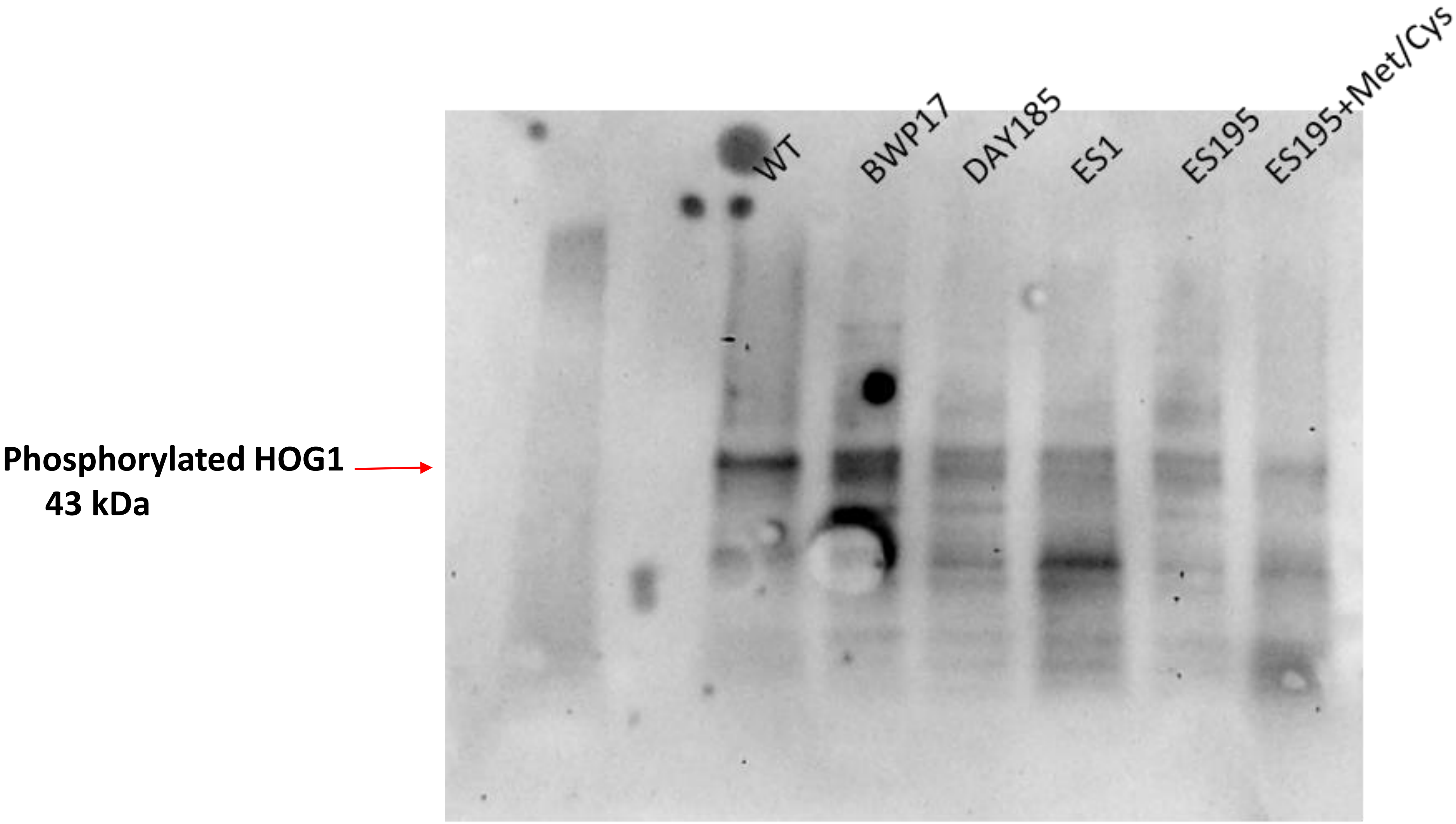

P-HOG1p
